# PlzA is a bifunctional c-di-GMP biosensor that promotes tick and mammalian host-adaptation of *Borrelia burgdorferi*

**DOI:** 10.1371/journal.ppat.1009725

**Published:** 2021-07-15

**Authors:** Ashley M. Groshong, André A. Grassmann, Amit Luthra, Melissa A. McLain, Anthony A. Provatas, Justin D. Radolf, Melissa J. Caimano

**Affiliations:** 1 Department of Medicine, UConn Health, Farmington, Connecticut, United States of America; 2 Department of Pediatrics, UConn Health, Farmington, Connecticut, United States of America; 3 Center for Environmental Sciences and Engineering, University of Connecticut, Storrs, Connecticut, United States of America; 4 Department of Molecular Biology and Biophysics, UConn Health, Farmington, Connecticut, United States of America; 5 Department of Genetics and Genome Science, UConn Health, Farmington, Connecticut, United States of America; 6 Department of Immunology, UConn Health, Farmington, Connecticut, United States of America; Medical College of Wisconsin, UNITED STATES

## Abstract

In this study, we examined the relationship between c-di-GMP and its only known effector protein, PlzA, in *Borrelia burgdorferi* during the arthropod and mammalian phases of the enzootic cycle. Using a *B*. *burgdorferi* strain expressing a *plzA* point mutant (*plzA-R145D*) unable to bind c-di-GMP, we confirmed that the protective function of PlzA in ticks is c-di-GMP-dependent. Unlike Δ*plzA* spirochetes, which are severely attenuated in mice, the *plzA-R145D* strain was fully infectious, firmly establishing that PlzA serves a c-di-GMP-independent function in mammals. Contrary to prior reports, loss of PlzA did not affect expression of RpoS or RpoS-dependent genes, which are essential for transmission, mammalian host-adaptation and murine infection. To ascertain the nature of PlzA’s c-di-GMP-independent function(s), we employed infection models using (i) host-adapted mutant spirochetes for needle inoculation of immunocompetent mice and (ii) infection of *scid* mice with *in vitro*-grown organisms. Both approaches substantially restored Δ*plzA* infectivity, suggesting that PlzA enables *B*. *burgdorferi* to overcome an early bottleneck to infection. Furthermore, using a *Borrelia* strain expressing a heterologous, constitutively active diguanylate cyclase, we demonstrate that ‘ectopic’ production of c-di-GMP in mammals abrogates spirochete virulence and interferes with RpoS function at the post-translational level in a PlzA-dependent manner. Structural modeling and SAXS analysis of liganded- and unliganded-PlzA revealed marked conformational changes that underlie its biphasic functionality. This structural plasticity likely enables PlzA to serve as a c-di-GMP biosensor that in its respective liganded and unliganded states promote vector- and host-adaptation by the Lyme disease spirochete.

## Author summary

Herein, we examined the roles of c-di-GMP and PlzA throughout the *Borrelia burgdorferi* lifecycle. Using a *plzA* point mutant that is unable to bind c-di-GMP, we confirmed that the protective function(s) of PlzA in feeding ticks and mammals are c-di-GMP-dependent and -independent, respectively. Contrary to previous studies, loss of PlzA did not affect expression of *rpoS* or RpoS-dependent genes, which are important for host-adaptation and persistence in the mammal. Host-adaptation prior to needle-inoculation of C3H mice or infection of *scid* mice substantially restored infectivity in the absence of PlzA. Using a *Borrelia* strain that synthesizes c-di-GMP constitutively, this secondary messenger was shown to be tick-phase specific and detrimental to host-adaptation *via* PlzA-mediated antagonism of RpoS function. Structural modeling and SAXS analysis of liganded- and unliganded-PlzA provide insight into the dramatic conformational changes upon c-di-GMP binding that likely underlie its differential function: liganded-PlzA supports survival within feeding ticks, while unliganded-PlzA promotes an RpoS-independent facet of host adaptation, enabling spirochetes to evade early immune clearance by the host.

## Introduction

Lyme disease, a multisystem disorder characterized by cutaneous, neurologic, cardiac, and rheumatologic manifestations [1,2], is caused by the spirochete *Borrelia burgdorferi* [3]. With ~35,000 confirmed cases reported to the Centers for Disease Control and Prevention annually [4,5], Lyme disease is the most prevalent arthropod-borne infection in the United States [6]. Based on insurance claims data, Kugeler *et al*. [5] estimated that during 2010 to 2018 the incidence of Lyme disease was ≈476,000 cases per year.

In nature, *B*. *burgdorferi* cycles between a hard tick vector and a vertebrate reservoir host, usually small rodents [3,7–9]; the generalist feeding behavior of *Ixodes* spp. is responsible for transmission of *B*. *burgdorferi* to humans, an incidental host [10,11]. Because transmission of *B*. *burgdorferi* is transstadial, larvae must acquire spirochetes by feeding on an infected reservoir host. Following acquisition, spirochetes enter a quiescent state within the midguts of flat nymphs [3,12]. The subsequent nymphal blood meal stimulates a replicative burst during which spirochetes replicate exponentially, traverse the midgut epithelium, migrate through the hemocoel to the salivary glands, and, following penetration of salivary acini, access the next host via the salivary stream [7,13,14]. To maintain its complex bi-phasic life cycle, *B*. *burgdorferi* must coordinate the expression of colonization factors and protective surface molecules and adjust its physiologic state to contend with vastly different environmental threats and nutrient profiles encountered in mammals and arthropods [3,15,16].

Two-component systems (TCS) are important mechanisms by which bacteria can adapt globally to their surroundings [17,18]. *B*. *burgdorferi* encodes only two TCSs, Hk1/Rrp1 and Hk2/Rrp2 [3,19,20]. While the sensory function(s) of Hk2 remains unclear [21,22], Rrp2 is part of a regulatory pathway in which the alternative sigma (σ^54^) factor RpoN transcribes the ‘effector’ alternative σ factor RpoS (σ^38^) [3,20,23–25]. Transcription of *rpoS* by Rrp2/RpoN also requires binding by the *B**orrelia* oxidative stress Regulator (BosR), which is a member of the Ferric Uptake Regulator (FUR) superfamily [20,26–32]. Genes within the RpoS regulon are required for tick transmission as well as to establish and sustain infection within the reservoir host [23,25,33–36]. The *hk1/rrp1* TCS encodes a membrane-associated hybrid sensory histidine kinase and response regulator with guanylate cyclase activity, respectively [37–39]. Upon binding of as yet unidentified exogenous ligand(s) (*i*.*e*., amino acids or their derivatives [40]), Hk1 initiates a signal transduction cascade that culminates in phosphorylation of Rrp1 and synthesis of bis-(3′-5′)-cyclic dimeric guanosine monophosphate (c-di-GMP) [37–39,41–46]. Spirochetes lacking either Hk1 or Rrp1 host-adapt normally within dialysis membrane chambers (DMCs) and are fully virulent in mice but are destroyed within feeding larvae and nymphs [38,39, 41–43,47].

Production of c-di-GMP has pleiotropic effects in Lyme disease spirochetes, affecting both motility and gene expression [38,39,42–45,48–53]. With respect to the latter, it promotes utilization of alternate carbon sources (*i*.*e*., glycerol, chitobiose and N-acetylglucosamine), upregulation of surface lipoproteins to ward off complement-mediated lysis, and protection against osmotic stress during tick feeding [38,41–43,45,54]. While we observed only limited overlap between the RpoS and Hk1/Rrp1 regulons *in vitro* [42], there is evidence for some degree of “cross-talk” between these two regulatory pathways. For example, within mammals, RpoS represses the expression of c-di-GMP-upregulated glycerol uptake and metabolism (*glp*) operon, which is essential for spirochete fitness in ticks [36,41,42,45,53,55,56]. Transcriptomic analyses of RpoS- and Rrp1-deficient spirochetes also identified genes that are upregulated by both regulatory pathways *in vitro* [38,41,42]. There are conflicting data, however, regarding the influence of c-di-GMP on expression of *ospC*, the prototypical RpoS-upregulated borrelial gene [38,41,42,45].

Efforts to elucidate how c-di-GMP exerts it effector function(s) in *B*. *burgdorferi* to date have centered about PlzA (BB0733), the sole PilZ-domain containing protein in strain B31 [42,44,46,49,51,53]. PlzA is monomeric and undergoes structural rearrangements upon binding of c-di-GMP to its PilZ domain *via* RxxxR and (D/N)*h*SxxG motifs [44,46,51]. As with Hk1/Rrp1 [39,41,42,47], PlzA has been shown to modulate expression of the *glp* operon *in vitro* [42,53] and spirochete survival in ticks [49,57]. Consistent with these data, expression of *plzA* is increased during tick feeding [46]. Paradoxically, in mice, an environment that does not stimulate Hk1/Rrp1-dependent synthesis of c-di-GMP, infectivity of PlzA-deficient organisms is markedly attenuated [49], an observation that contrasts with findings that Δ*hk1* and Δ*rrp1* strains are fully virulent [39,41–43]. The reduced virulence of Δ*plzA* spirochetes in mice has been attributed to abnormal growth and motility, altered cell envelope biogenesis and/or decreased levels of BosR [45,49,52,57]. Collectively, these studies point to multiple effector functions and, likely, different interaction partners for liganded- and unliganded-PlzA in ticks and mice, respectively [49,53,57].

The unresolved issues surrounding the relationship between PlzA, c-di-GMP signaling and RpoS-dependent gene regulation prompted us to re-examine the role of this novel c-di-GMP effector throughout the enzootic cycle. Herein, we establish unequivocally that PlzA function in ticks is c-di-GMP-dependent. Conversely, binding of c-di-GMP by PlzA is not required by *B*. *burgdorferi* to establish or sustain infection in mice following needle-inoculation. Unliganded-PlzA, however, promotes an RpoS-independent facet of mammalian host adaptation that enables spirochetes to overcome an early infection bottleneck. Using a *B*. *burgdorferi* strain that synthesizes c-di-GMP constitutively at measurable amounts *in vitro*, we show that the presence of this second messenger is detrimental to murine infectivity due, at least in part, to PlzA-mediated antagonism of RpoS function at the post-translational level. Using small angle X-ray scattering (SAXS) analysis and computational modeling, we generated the first three-dimensional structural model for liganded-PlzA. Consistent with FRET studies by Mallory *et al*. [51], SAXS analysis revealed substantial conformational changes in PlzA upon c-di-GMP binding. PlzA’s structural plasticity likely underlies its ability to serve as both a c-di-GMP biosensor within feeding ticks and a c-di-GMP-independent regulatory protein in mammals.

## Results

### Protection of spirochetes by PlzA during tick feeding is c-di-GMP-dependent

Prior studies by us [36,39] and others [38,41,43,49,57] demonstrating that both the Hk1/Rrp1 TCS and PlzA are required for survival of *B*. *burgdorferi* during the blood meal strongly suggest that PlzA functions as a c-di-GMP biosensor in feeding ticks. To confirm this experimentally, we took advantage of a finding by Mallory *et al*. [51] demonstrating that recombinant PlzA containing an arginine to aspartic acid substitution at residue 145 (R145D), the first position of the RxxxR motif, completely abolished c-di-GMP binding. At the outset, we confirmed this finding by comparing the ability of recombinant wild-type and PlzA-R145D His-tagged proteins to bind a fluorescent c-di-GMP analog (2’-O-(N’-methylanthraniloyl)-cyclic diguanosine monophosphate [MANT-c-di-GMP]) [58] (S1A Fig). As shown in S1B Fig, at the emission wavelength of 450 nm (MANT λem_max_ = 448 nm), PlzA-R145D and the lysozyme negative control bound significantly less (*p* < 0.0001) MANT-c-di-GMP than wild-type PlzA.

**Fig 1:**
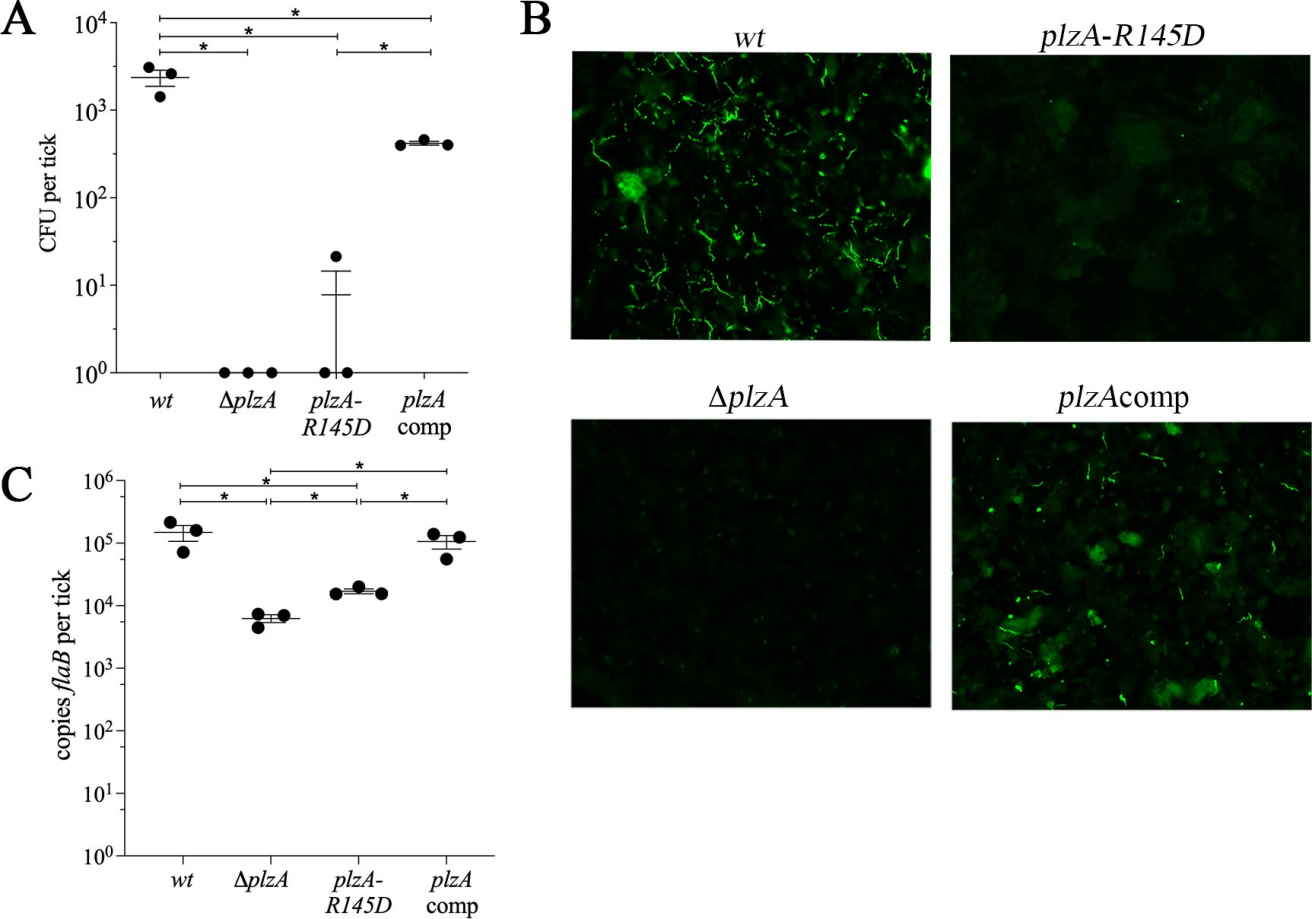
Binding of c-di-GMP by PlzA is essential for survival of *B*. *burgdorferi* during tick feeding. (**A**) Viable spirochete burdens for larvae immersion-fed with B31 A3-68 Δ*bbe02* (*wt*), Δ*plzA*, *plzA-R145D* and *plzA*comp strains as determined by colony forming units (CFU). (**B**) Representative immunofluorescence images of immersion-fed larvae using FITC-conjugated anti-*Borrelia* antibody. (**C**) Burdens in immersion-fed larvae determined by qPCR using a TaqMan assay for *flaB*. Data points in panels **A** and **C** represent individual pools of larvae. Error bars indicate the mean ± standard error of the mean for each strain normalized per tick. Asterisks (*) indicate statistical significance (*p* < 0.05) of all pairwise comparisons determined by unpaired Student’s *t*-test.

To evaluate the consequence of the R145D substitution on PlzA function in ticks, we inserted the mutated allele into the native *plzA* locus of BbP1473, a wild-type (*wt*) strain B31 A3-68 isolate (S1 Table). The resulting mutant, designated *plzA-R145D*, was tested in parallel with isogenic *wt*, Δ*plzA* and *plzA* complemented (*plzA*comp) strains in larvae infected *via* immersion and then fed to repletion on naïve C3H/HeJ mice as previously described [39,42]. In all comparisons, the *plzA-R145D* mutant was indistinguishable from its Δ*plzA* counterpart. By semi-solid phase plating, no viable *plzA-R145D* or Δ*plzA* spirochetes were recovered from replete larvae (Fig 1A). Immunofluorescence assay (IFA) of larval midguts revealed only sparse spirochete remnants for both of the PlzA mutant strains (Fig 1B). By qPCR, we detected ~1-log_10_ lower burdens for the *plzA-R145D* and Δ*plzA* mutants in replete larvae compared to the *wt* and *plzA*comp strains (Fig 1C). Based on prior studies [39,41,42,45,54] and the IFA results herein, we attribute the decreased spirochete burdens for both mutants to their destruction during tick feeding. Collectively, these data establish unequivocally that the protection afforded by PlzA during the blood meal is c-di-GMP-dependent.

### Loss of PlzA markedly impairs spirochete infectivity in a c-di-GMP-independent manner

Unlike Δ*hk1* and Δ*rrp1* strain, which display *wt* infectivity in mice [39,41,42], Δ*plzA* organisms are highly attenuated [49,57]. Thus, while the phenotypes for all three mutants are highly similar, if not identical, in ticks (Fig 1), their phenotypes in mice are dichotomous. Importantly, these data suggest that PlzA function in mammals is c-di-GMP-independent. To garner support for this notion, we compared infectivity of *plzA-R145D* and *plzA* strains in C3H/HeJ mice in parallel with *wt* and *plzA*comp strains. Consistent with prior studies [49,57], infectivity of the Δ*plzA* mutant was markedly impaired compared to *wt* at two-weeks post-infection (Table 1). Of the ten mice infected with the Δ*plzA* strain, only two seroconverted (S2 Fig). Though not every tissue from the two Δ*plzA*-infected mice was culture positive (Table 1), in these animals, the mutant clearly disseminated from the site of inoculation. In contrast, all mice infected with the *plzA-R145D* mutant seroconverted and nearly all tissues were culture-positive at two-weeks post needle-inoculation (Table 1 and S2 Fig), thereby confirming that PlzA function in the mammal does not require binding of c-di-GMP. Complementation restored infectivity of the Δ*plzA* strain to *wt* levels (Table 1).

As noted earlier, attenuation of PlzA-deficient strains has been attributed to abnormal growth and/or motility *in vitro* in standard BSK medium [49,57]. However, in side-by-side growth curves with *wt*, Δ*rrp1*, Δ*plzA* and *plzA*comp strains, the virulent *plzA-R145D* mutant exhibited a more pronounced growth defect than the attenuated Δ*plzA* null mutant (*p* = 0.025) (S3 Fig). Moreover, we saw no significant difference in growth between Δ*rrp1* spirochetes, which are fully virulent ([41,42] and below), and the Δ*plzA* mutant (S3 Fig). Thus, while the Δ*plzA* strain grows more slowly than its *wt* and *plzA*comp counterparts *in vitro* (*p*<0.05), this phenotype does not explain its markedly reduced virulence in mice. By darkfield microscopy, we also compared the motility of *wt*, Δ*rrp1* and Δ*plzA* strains in BSK-II medium. As previously reported [43], Rrp1-deficient spirochetes display a faster run speed and significantly decreased flexing compared to *wt*, essentially locking them in “run” mode (S1 and S2 Movies). In contrast, the swimming behaviors of *wt* and Δ*plzA* (S1 and S3 Movies, respectively) were indistinguishable, as previously noted [49,57].

**Table 1:** c-di-GMP binding by PlzA is not required for murine infectivity.

	*wt*^2^	Δ*plzA*^2^	*plzA-R145D*	*plzA*comp
**Serology**^**1**^	10/10	2/10	5/5	5/5
**Ear**^**3**^	10/10	0/10	5/5	5/5
**Proximal skin**	10/10	2/10	5/5	5/5
**Distal skin**	10/10	2/10	5/5	4/5
**Tibiotarsal joint**	10/10	1/10	4/5	5/5
**Bladder**	10/10	2/10	5/5	5/5
**Heart**	10/10	2/10	5/5	5/5
**Total positive sites**^**4**^	60/60	9/60	29/30	29/30
**Total mice infected**^**5**^	10/10	2/10	5/5	5/5

^1^Serology is based on immunoreactivity of serum from individual mice against whole cell lysates of wild-type *B*. *burgdorferi* strain B31 cultivated at 37°C *in vitro*. Immunoblot data for individual mice are presented in S2 Fig.

^2^Wild-type and Δ*plzA* strains were compared to *plzA-R145D* and *plzA*comp strains in separate experiments (5 mice per strain, per experiment).

^3^Data represent culture positivity for the designated tissues collected from C3H/HeJ mice two weeks after inoculation with 1 × 10^4^ of wild-type (*wt*), Δ*plzA*, *plzA-R145D* or *plzA*comp strains cultivated *in vitro*.

^4^Total number of culture-positive tissues from all mice in the designated group.

^5^Total number of infected mice per group.

### Attenuation of Δ*plzA* spirochetes is RpoS-independent

**Fig 2:**
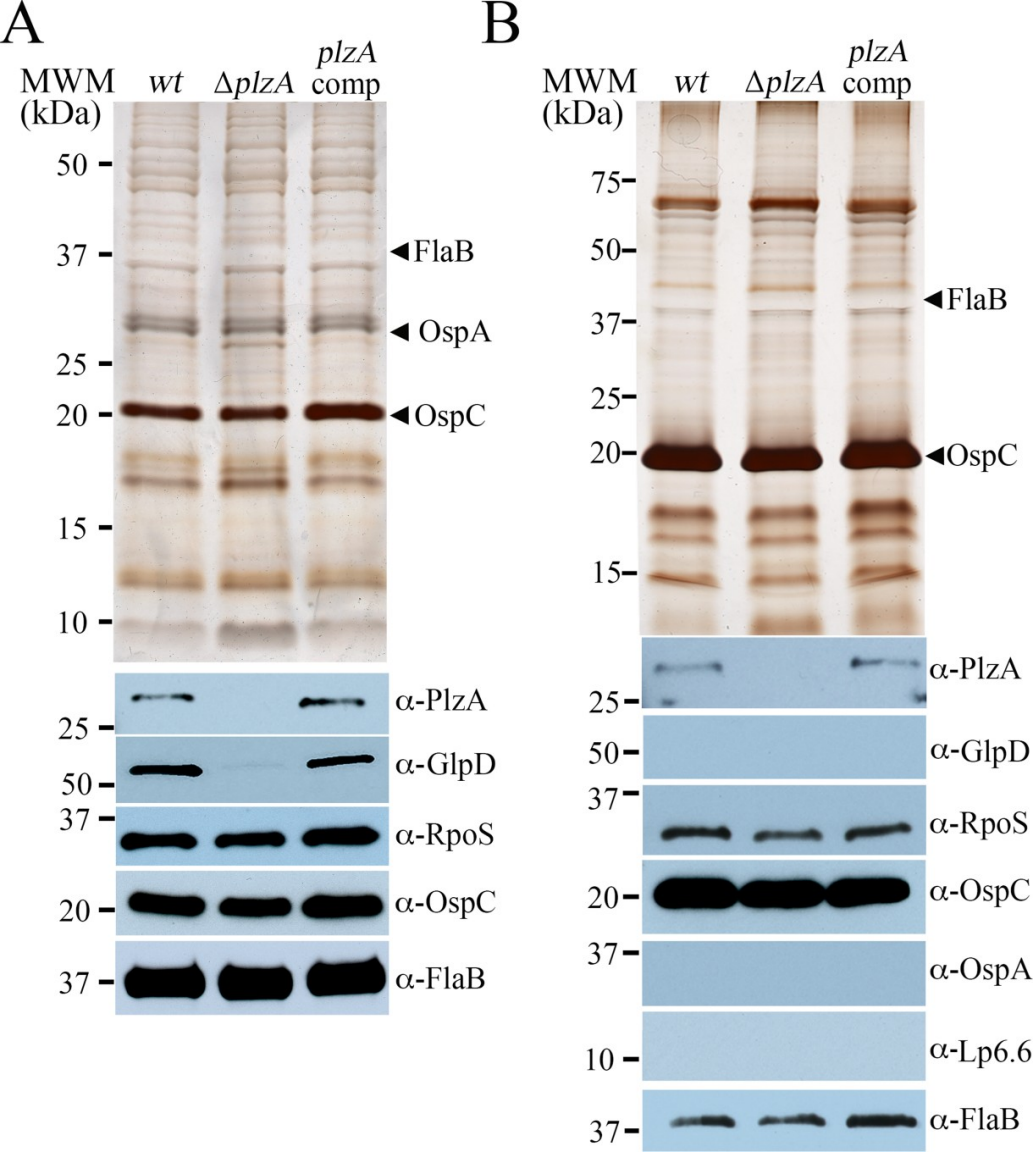
PlzA deficiency does not impair the RpoN/RpoS pathway *in vitro* or during cultivation in DMCs. Whole-cell lysates from B31 A3-68 Δ*bbe02* (*wt*), Δ*plzA* and *plzA*comp strains following temperature-shift *in vitro* (**A**) or cultivation within rat peritoneal dialysis membrane chambers (**B**) were separated by SDS-PAGE and stained with silver or immunoblotted using antisera against PlzA, GlpD, RpoS, OspC, OspA, Lp6.6 or FlaB (loading control). Molecular weight markers (MWM) are shown on the left.

The reduced virulence of PlzA-deficient spirochetes has been attributed to loss of BosR expression with subsequent ablation of the RpoN/RpoS pathway [52]. In our hands, however, *wt*, Δ*plzA* and *plzA*comp strains express comparable levels of RpoS and OspC, the prototypical RpoS-dependent gene product, *in vitro* following temperature-shift (Fig 2A). As a control, lysates were immunoblotted for GlpD, a known c-di-GMP-dependent downstream target of PlzA [36,41,55,56]. Because *in vitro* cultivation does not recapitulate the full spectrum of transcriptional changes associated mammalian-host specific signals [36,55,59,60], we examined whether loss of PlzA impaired RpoS-dependent function(s) that are specific to mammalian host-adaptation. Fig 2B shows that this is not the case. In spirochetes lacking PlzA, RpoS and OspC were produced at essentially *wt* levels, while RpoS-repressed tick-phase lipoproteins OspA and BBA62/Lp6.6 were appropriately downregulated [36,55,61–63].

### PlzA overcomes an RpoS-independent immune bottleneck during early infection

To examine whether host-adaptation prior to needle-inoculation enables spirochetes to overcome the early infection defect caused by loss of PlzA, *wt*, Δ*plzA*, *plzA-R145D* and *plzA*comp strains were cultivated in DMCs and then immediately used to inoculate (1 × 10^4^ spirochetes) naïve C3H/HeJ mice. Analysis of whole cell lysates confirmed that all strains had properly host-adapted prior to inoculation (S4A Fig). In contrast to mice inoculated with *in vitro*-cultivated Δ*plzA* organisms (Table 1), all mice needle-inoculated with DMC-cultivated Δ*plzA* organisms seroconverted (Table 2 and S4B Fig) and were culture positive at three or more tissue sites at two weeks post-infection (Table 2). As expected, *plzA-R145D* and *plzA*comp strains displayed infectivity comparable to that of the *wt* parent (Table 2). To determine whether adaptive immune pressure contributes to the attenuation of the Δ*plzA* mutant, we assessed infectivity of *wt*, Δ*plzA* and *plzA*comp strains (1 × 10^4^ spirochetes) in immunodeficient Prkdc^scid^ (*scid*) mice four-weeks post-inoculation. As shown in Table 3, the Δ*plzA* strain infected *scid* mice at near *wt* levels. Collectively, these data suggest that PlzA promotes the expression of one or more virulence-related gene products required to evade host adaptive immunity.

**Table 2:** Prior host-adaptation enhances infectivity of PlzA-deficient spirochetes.

	*wt*^1^	Δ*plzA*^1^	*plzA-R145D*	*plzA*comp
**Serology**^**2**^	5/5	9/9	3/3	3/3
**Ear**^**3**^	5/5	4/9	2/3	3/3
**Proximal skin**	5/5	8/9	3/3	3/3
**Distal skin**	5/5	8/9	3/3	3/3
**Tibiotarsal joint**	5/5	4/9	3/3	2/3
**Bladder**	5/5	8/9	3/3	3/3
**Heart**	5/5	9/9	3/3	3/3
**Total positive sites**^**4**^	30/30	41/54	17/18	17/18
**Total mice infected**^**5**^	5/5	9/9	3/3	3/3

^1^Wild-type and Δ*plzA* strains were compared to *plzA-R145D* and *plzA*comp strains in separate experiments.

^2^Serology is based on immunoreactivity of serum from individual mice against whole cell lysates of wild-type *B*. *burgdorferi* strain B31 cultivated at 37°C *in vitro* (S4B Fig).

^3^Data represent culture positivity for the designated tissues collected from C3H/HeJ mice two weeks after inoculation with 1 × 10^4^ of wild-type (*wt*), Δ*plzA*, *plzA-R145D* or *plzA*comp strains cultivated in DMCs.

^4^Total number of culture-positive tissues from all mice in the designated group.

^5^Total number of infected mice per group.

**Table 3:** PlzA-deficient spirochetes show increased infectivity in immunodeficient (*scid*) mice.

	*wt*	Δ*plzA*	*plzA*comp
Ear^1^	3/3	3/3	3/3
**Proximal skin**	3/3	3/3	3/3
**Distal skin**	3/3	2/3	3/3
**Tibiotarsal joint**	3/3	1/3	3/3
**Bladder**	3/3	3/3	3/3
**Heart**	3/3	1/3	3/3
**Total positive sites**^**2**^	18/18	13/18	18/18
**Total mice infected**^**3**^	3/3	3/3	3/3

^1^Data represent culture positivity for the designated tissues collected from NOD.Cg-*Prkdc*^*scid*^/J (*scid*) mice (3 per group) four weeks after inoculation with 1 × 10^4^ of wild-type (*wt*), Δ*plzA* or *plzA*comp strains cultivated *in vitro*.

^2^Total number of culture-positive tissues from all mice in the designated group.

^3^Total number of infected mice per group.

### Constitutive production of c-di-GMP ablates spirochete virulence

**Fig 3:**
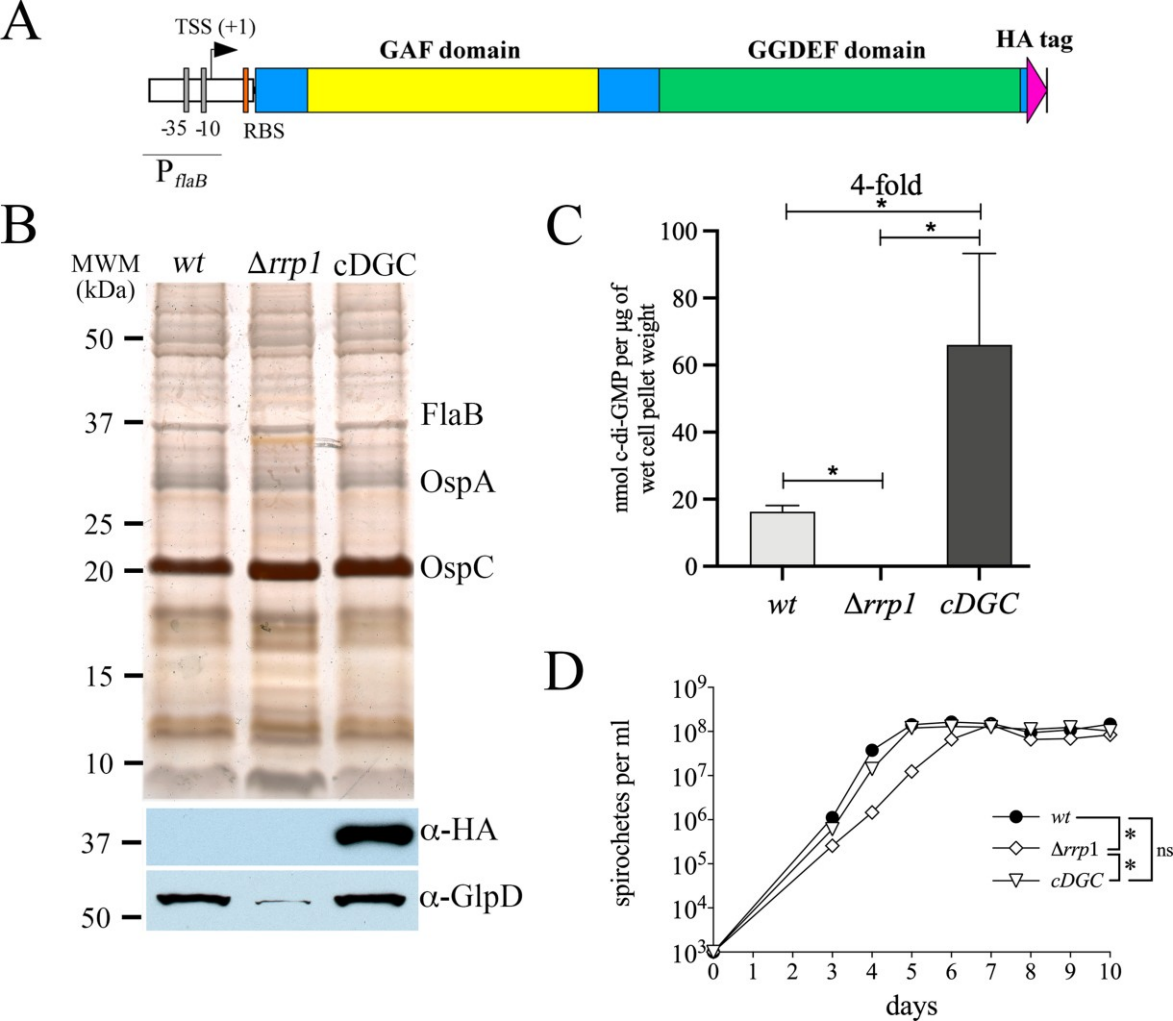
Constitutive synthesis of c-di-GMP functionally complements GlpD expression and motility in Δ*rrp1* spirochetes *in vitro*. (**A**) Cartoon depiction of the P_*flaB*_-*slr1143*-HA construct encoding a *B*. *burgdorferi* codon-optimized, constitutively active Slr1143 diguanylate cyclase (cDGC) from *Synechocystis* sp. [85]. TSS (+1), transcriptional start site; P*flaB*, *flaB* promoter; RBS, ribosome binding site; HA, hemagglutinin tag. (**B**) Whole-cell lysates of B31 5A18 NP1 (*wt*), Δ*rrp1* and *cDGC* strains grown *in vitro* following temperature-shift were separated by SDS-PAGE and stained with silver or immunoblotted with antibodies against HA or GlpD. Molecular weight markers (MWM) are shown on the left. (**C**) c-di-GMP measurements for B31 5A18 NP1 (*wt*), Δ*rrp1*, and c*DGC* strains as determined by LC-MS/MS. Bars represent the mean ± standard error of the mean for three independent cultures per strain. Statistical significance was determined by unpaired Student’s *t*-test. Asterisks (*) indicate statistical significance (*p* ≤ 0.05) of all pairwise comparisons. (**D**) Growth curves of 5A18 NP1 (*wt*), Δ*rrp1* and *cDGC* strains (in quadruplicate) from a starting density of 1 × 10^3^ spirochetes per ml at 37°C. Statistical significance was determined using the CGGC permutation test [122]. Asterisks (*) indicate statistical significance (*p* ≤ 0.05) of all pairwise comparisons; ns, not significant.

**Fig 4:**
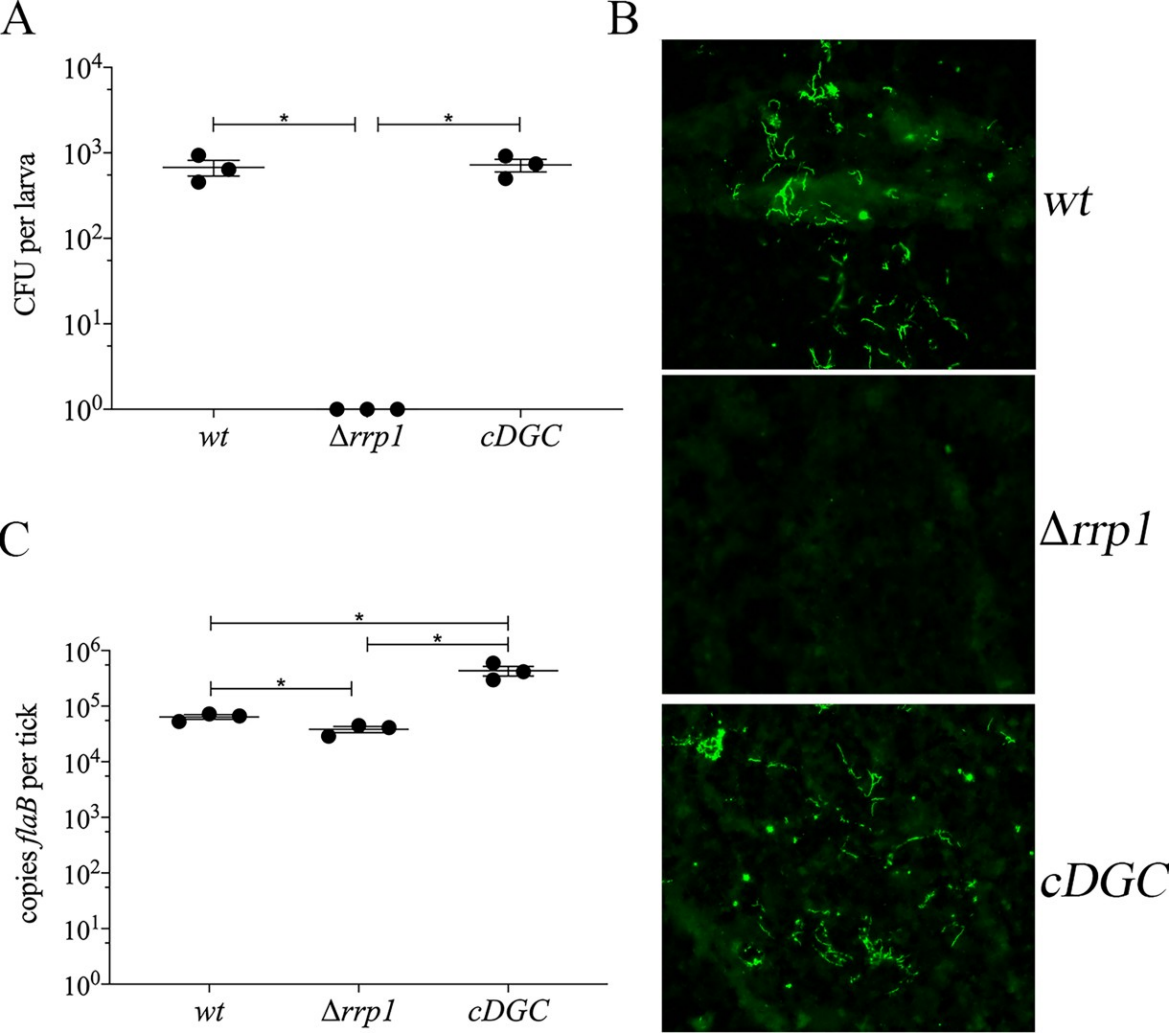
Constitutive synthesis of c-di-GMP functionally complements Δ*rrp1* in feeding ticks. (**A**) Viable spirochete burdens in larvae immersion-fed with B31 5A18 NP1 wild-type (*wt*), Δ*rrp1* and *cDGC* strains as determined by colony forming units (CFUs) following semi-solid plating. (**B**) Representative immunofluorescence images of immersion-fed larvae using FITC-conjugated anti-*Borrelia* antibody. (**C**) DNA burdens in immersion fed larvae determined by qPCR using a TaqMan assay for *flaB*. Data points in panels A and C represent individual pools of ticks. Error bars indicate the mean ± standard error of the mean for each strain normalized per tick. Asterisks (*) indicate statistical significance (*p* ≤ 0.05) of all pairwise comparisons, as determined by unpaired Student’s *t*-test.

Kostick *et al*. [43] previously reported that overexpression of Rrp1 in a wild-type *B*. *burgdorferi* background had no effect on motility or chemotaxis *in vitro* but substantially attenuated virulence in mice infected by needle-inoculation. These results suggest that c-di-GMP is deleterious to spirochetes during the mammalian host phase of the enzootic cycle. However, because the authors were unable to measure c-di-GMP levels in either the wild-type or Rrp1-overexpressing strains, it is unclear how the levels of this secondary messenger compare in in the two strains. Thus, to further investigate the effect of c-di-GMP on infectivity in the mammal, we took advantage of studies by Ryjenkov *et al*. [37] demonstrating that Slr1143, a diguanylate cyclase from an oxygenic phototroph *Synechocystis* sp., constitutively synthesizes c-di-GMP. As described in Methods, we generated a cp26-based suicide-vector containing a *Borrelia*-codon-optimized, hemagglutinin (HA)-tagged *slr1143* construct expressed under the constitutive borrelial *flaB* promoter (Fig 3A) and transformed it into a strain B31 Δ*rrp1* mutant. Constitutive expression of HA-tagged Slr1143 in this strain (designated *cDGC*) was confirmed by immunoblot using anti-HA antibodies (Fig 3B). *cDGC* grown at 37°C to late-logarithmic phase harbored concentrations of c-di-GMP, measured by LS-MS/MS, only slightly (3-4-fold) greater than those in the *wt*; as expected, no c-di-GMP was detected in the Δ*rrp1* control (Fig 3C). During *in vitro* growth, constitutive synthesis of c-di-GMP by Slr1143 functionally complemented loss of Rrp1 based on restoration of GlpD expression (Fig 3B), *wt* growth kinetics (Fig 3D), and normal motility (S4 Movie). Lastly, we examined the survival of the *cDGC* strain in ticks (Fig 4). As expected [39,41–43,45], and similar to the Δ*plzA* and *plzA-R145D* mutant phenotypes (Fig 1), the Δ*rrp1* mutant did not survive the blood meal (Fig 4A and 4B), though qPCR detected residual spirochete DNA (Fig 4C). Importantly, Slr1143 functionally complemented the Δ*rrp1* mutation, restoring the mutant’s ability to survive during the larval blood meal (Fig 4).

We next used the *cDGC* strain to assess how continued synthesis of c-di-GMP affects the ability of Lyme disease spirochetes to establish infection, disseminate and persist in mice. Naïve C3H/HeJ mice were needle-inoculated (1 × 10^4^ spirochetes) with *wt*, Δ*rrp1* and *cDGC* strains and infection was assessed by tissue culturing at two weeks post-inoculation. In contrast to mice infected with the *wt* and Δ*rrp1* strains, all of which were culture positive at each site tested, none of the mice inoculated with the *cDGC* strain yielded positive tissue cultures or seroconverted (S2 Table).

### Production of c-di-GMP antagonizes the RpoN/RpoS pathway through PlzA

**Fig 5:**
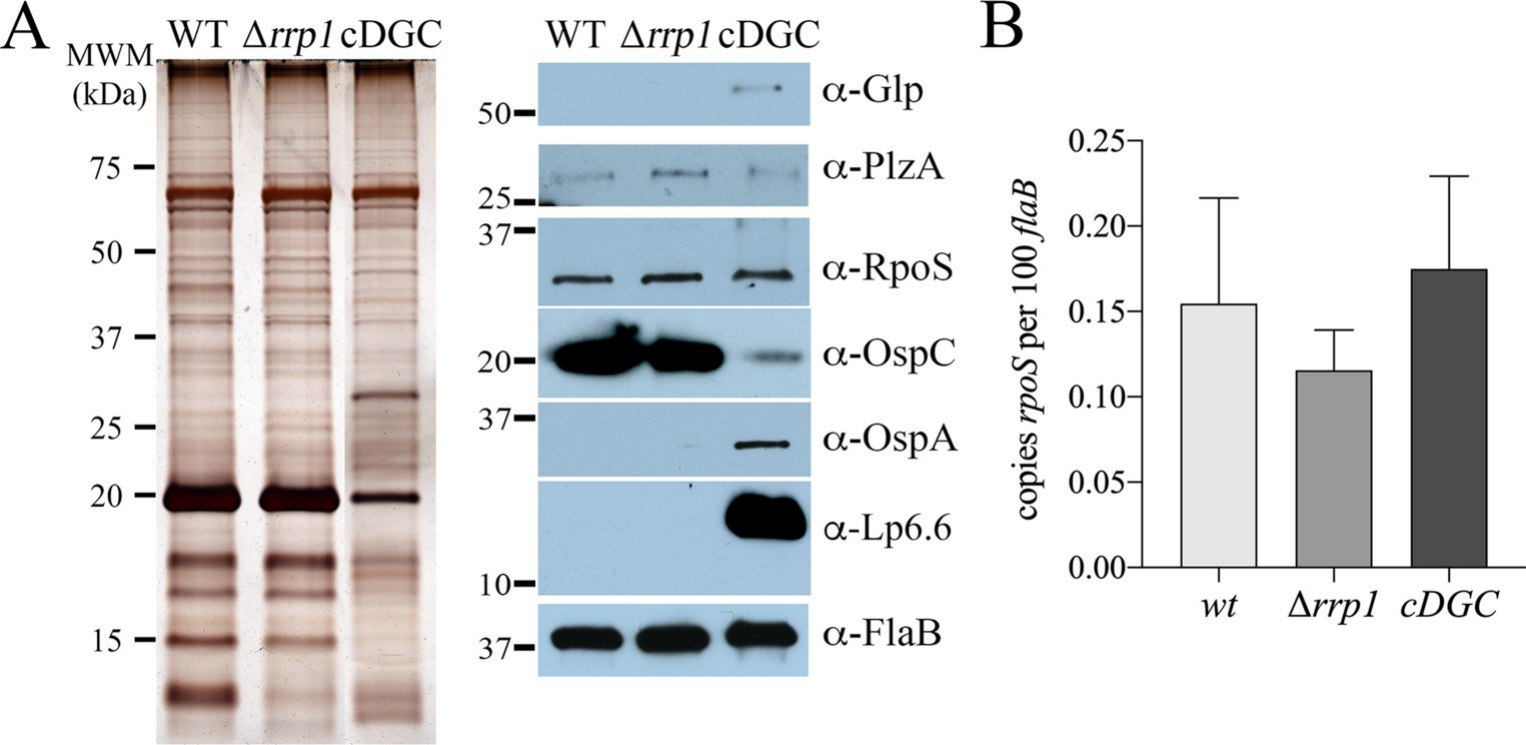
Constitutive synthesis of c-di-GMP antagonizes the RpoN/RpoS pathway in mammalian host-adapted spirochetes. (**A**) Whole-cell lysates from B31 5A18 NP1 wild-type (*wt*), Δ*rrp1*, and *cDGC* strains cultivated in DMCs were separated by SDS-PAGE and stained with silver or immunoblotted with antisera against GlpD, PlzA, RpoS, OspC, OspA, Lp6.6 and FlaB (loading control). Molecular weight markers (MWM) are shown on the left. (**B**) Expression of *rpoS* determined by qRT-PCR using RNA extracted from *wt*, Δ*rrp1* and *cDGC* strains cultivated in DMCs. Transcript copy numbers for *rpoS* were normalized using *flaB*. Statistical significance was determined by unpaired Student’s *t*-test. No significance difference (*p* ≥ 0.05) was observed for any pairwise comparison.

We postulated that antagonism of RpoS-dependent gene regulation by c-di-GMP might explain the avirulence of the *cDGC* strain. To investigate this possibility, we cultivated wild-type, Δ*rrp1* and *cDGC* strains in DMCs. Whereas *wt* and Δ*rrp1* spirochetes host-adapted normally, the *cDGC* strain demonstrated a markedly abnormal protein profile with significant reduction of OspC and strong expression of OspA and Lp6.6, both of which are RpoS-repressed during mammalian host adaptation [36,55,61] (Fig 5A). Notably, we also detected GlpD in the *cDGC* strain (Fig 5A), indicating that c-di-GMP is able to promote *glp* gene expression when RpoS-mediated repression of *glp* gene transcription [20,36,55,62] is antagonized. Surprisingly, by both immunoblot (Fig 5A) and qRT-PCR (Fig 5B), *rpoS*/RpoS levels in all three strains were equivalent in DMCs, confirming c-di-GMP interferes with RpoS function at the post-translational level. Of note, constitutive synthesis of c-di-GMP had no effect on OspC expression *in vitro*, suggesting this phenomenon is unique to host-adaptation (S5 Fig).

**Fig 6:**
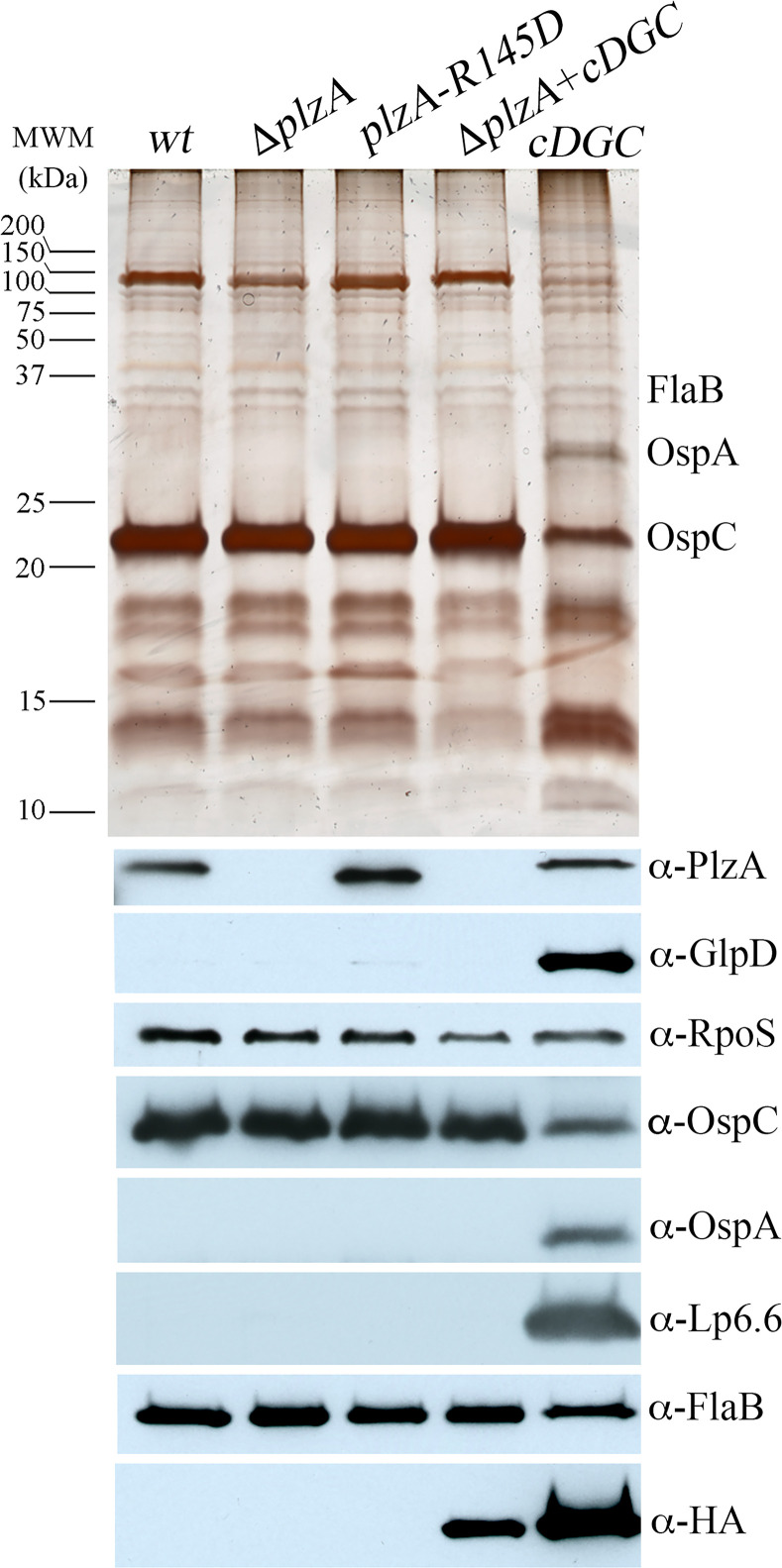
c-di-GMP requires PlzA to antagonize RpoS-dependent mammalian host-adaptation. Whole-cell lysates from wild-type B31 A3-68 Δ*bbe02* wild-type (*wt*), Δ*plzA*, *plzA-R145D*, Δ*plzA*+*cDGC* and *cDGC* strains cultivated within DMCs, separated by SDS-PAGE and stained with silver or immunoblotted with antisera against PlzA, GlpD, RpoS, OspC, OspA, Lp6.6, HA, and FlaB (loading control). Molecular weight markers (MWM) are shown on the left.

To determine whether the interference of c-di-GMP on RpoS function in DMCs is mediated *via* PlzA, we inserted the constitutive-expressed/-active diguanylate cyclase (P_*flaB*_-*slr1143-HA*) cassette into the Δ*plz* background, generating Δ*plzA+cDGC*, and evaluated the protein profiles of *wt*, Δ*plzA*, *plzA-R145D*, Δ*plzA+cDGC* and *cDGC* strains cultivated in DMCs by SDS-PAGE and immunoblot (Fig 6). Whereas the presence of c-di-GMP (*cDGC*) interfered with both positive and negative aspects of RpoS-dependent gene regulation in DMCs, the absence of PlzA (Δ*plzA+cDGC*) restored normal RpoS function *in vivo*. Consistent with results above (S4 Fig), *wt*, Δ*plzA* and *plzA-R145D* strains all host-adapted normally (Fig 6). From these data, we conclude that ectopic expression of c-di-GMP *in vivo* dampens RpoS activity at the post-translational level in a PlzA-dependent manner.

### PlzA has a flexible bipartite domain structure that potentially explains its differential function

Galperin and Chou [64] recently categorized bipartite ‘xPilZ-PilZ’ proteins from diverse bacteria into nine classes based on their N-terminal non-canonical PilZ-related domains. PlzA belongs to the ‘PilZN3-PilZ’ class based on the presence of a newly described N-terminal PilZN3 domain, which is predicted to form a six-stranded PilZ-like β-barrel. To date, crystal structures have been reported for only three xPilZ-PilZ proteins in c-di-GMP-liganded and -unliganded states (S6A Fig): (i) MrkH, a PilZN2-PilZ DNA binding transcriptional factor that promotes biofilm formation in *Klebsiella pneumoniae* [65,66]; (ii) FlgZ, a PilZN-PilZ protein, which functions as a flagellar brake in *Pseudomonas putida* [67]; and (iii) PlzD, a PilZNR-PilZ protein, which regulates virulence and motility in *Vibrio cholerae* [68,69]. In all three, binding of c-di-GMP by the C-terminal PilZ domain induces a large rotational change that brings their N- and C-terminal β-barrels into proximity, with c-di-GMP intercalated at xPilZ-PilZ domain interface. Root-mean-square deviation (RMSD) values for liganded and unliganded MrkH, FlgZ and PlzD (S6B Fig) confirm that the major conformational changes occur at the xPilZ-PilZ domain interface (11.8–13.0 Å), with essentially no structural changes in the individual β-barrels (0.2–0.5 Å).

**Fig 7:**
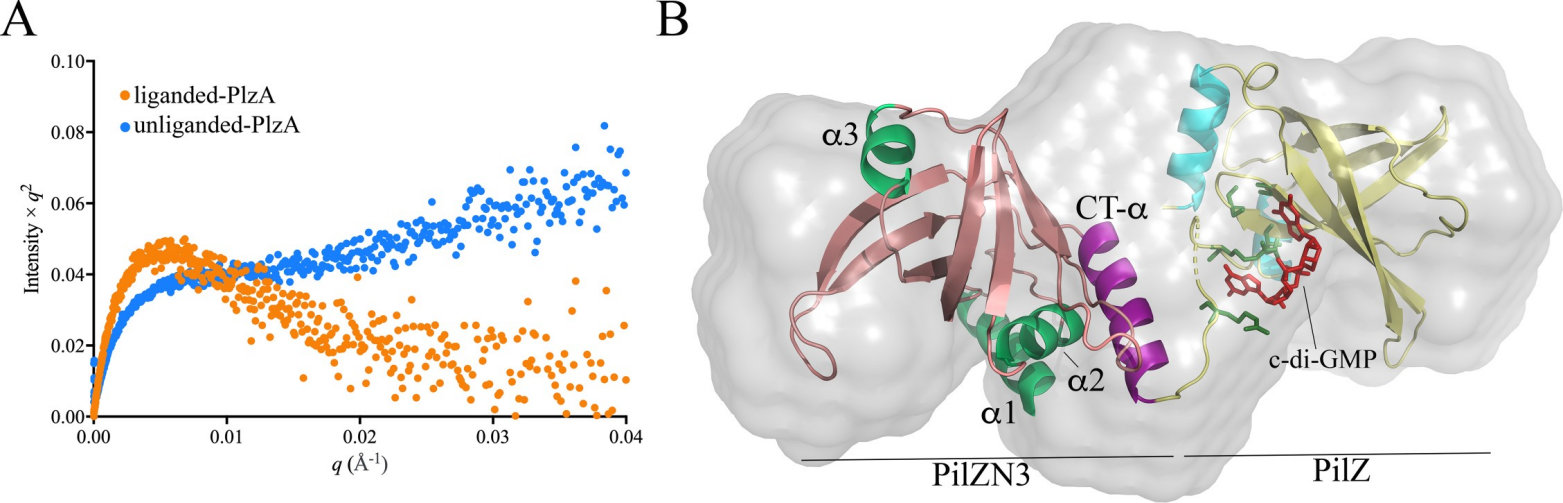
Structural analysis of PlzA. (**A**) Kratky plots for liganded (orange) and unliganded PlzA (blue). (**B**) Superposition of the SAXS envelope and refined structural model for liganded-PlzA. The PilZN3 and PilZ β-barrels are colored in salmon and yellow, respectively. The α helices in the PilZN3 and PilZ domains are highlighted in light green and cyan, respectively, while the C-terminal α-helix in PilZN3 is shown in purple. c-di-GMP binding residues in RXXXR and DXSXXG motifs are highlighted in dark green. c-di-GMP (red) was docked into the model using HADDOCK [75].

Using a FRET-based approach, Mallory *et al*. [51] demonstrated that recombinant PlzA undergoes ligand-induced rearrangement. To elucidate the structural changes that PlzA undergoes upon binding of c-di-GMP, we performed small angle X-ray scattering (SAXS) on recombinant monomeric His-tagged PlzA (S7A Fig) in the presence and absence of c-di-GMP (S7B Fig). Shapes of the Kratky plots (Fig 7A) revealed that liganded-PlzA is well-ordered, while the unliganded protein is unfolded and/or highly flexible, confirming a major structural rearrangement upon binding of c-di-GMP. We next used trRosetta [70], I-TASSER [71,72] and SWISS-MODEL [73] to generate structural models for liganded-PlzA and then screened twenty of the resulting models against our experimental SAXS data (S4 Table). One model, generated using trRosetta, produced the lowest χ^2^ value (7.14) which, after SREFLEX refinement [74], improved to 6.66 (S7C Fig). Of note, refinement did not require structural changes within the N- or C-terminal domains, just reorientation of the β-barrels. Next, the refined model was docked with c-di-GMP using HADDOCK [75] and fit into the SAXS envelope. As shown in Figs 6D and S7D, the RxxxR c-di-GMP binding motif is located within the extended interdomain linker, while the (D/N)*h*SxxG motif is located within the C-terminal PilZ β-barrel. Helices α1 and α2 in the N-terminal PilZN3 domain are positioned in proximity to its unique C-terminal α-helix (CT-α) and RxxxR motif. Electrostatics analysis of liganded-PlzA indicates three positively charged surface regions; one contains the c-di-GMP binding site, while the other two are located in grooves within the N- and C-terminal domains (S7E Fig, dashed lines) and could be available for interactions with DNA or other proteins. Due to its high ambiguity score, an envelope for unliganded PlzA could not be generated from the SAXS data, further confirming that c-di-GMP locks both domains of PlzA into a static, condensed conformation.

**Note added in proof:** Singh et al. [123] recently reported a 1.6 Å high resolution crystal structure for *Borrelia* (*Borreliella*) *burgdorferi* PlzA in complex with two molecules of c-di-GMP (PDB ID: 7MIE). The structural model and domain organization for PlzA presented herein are highly similar to the solved structure reported by Singh et al.

## Discussion

In many bacteria, c-di-GMP inhibits motility [76] and, by a variety of allosteric mechanisms, regulates the transition from a motile planktonic to a biofilm-associated, communal lifestyle [77–81]. In *B*. *burgdorferi*, c-di-GMP production is tied to environmental sensing by the Hk1/Rrp1 TCS in response to unknown biochemical cues generated during tick feeding [38–44]. Spirochetes unable to produce c-di-GMP cannot withstand the onslaught of nutritional and/or biophysical stressors unleashed by the blood meal and, consequently, are destroyed in the midguts of both larvae and nymphs [39,41–43]. Since the signals driving Hk1/Rrp1 activation emanate from the blood meal [38–43], production of and signaling by c-di-GMP is restricted to the tick phase of the enzootic cycle. Indeed, not only is c-di-GMP not required during the reservoir phase [39,41,42], herein, we present evidence that it is inimical, at least in part, because it antagonizes RpoS function(s) required for the establishment and maintenance of mammalian infection by *B*. *burgdorferi*. A conundrum the spirochete faces is that it must produce c-di-GMP during two ‘opposing’ tick phases—acquisition, the RpoS-OFF state when spirochetes colonize the vector, and transmission, the RpoS-ON state when spirochetes regain motility and exit the midgut [20,34,35,42,55,82]. *B*. *burgdorferi* appears to have partially resolved this dilemma by integrating the adaptive changes mediated by c-di-GMP-liganded PlzA into the ON and OFF states of the RpoS genetic program in ticks along with divergent motility responses during the acquisition and transmission blood meals. Moreover, in PlzA, the spirochete has evolved a novel c-di-GMP effector protein [64] that serves as a biosensor for the presence or absence of c-di-GMP to promote, respectively, vector- or mammalian host-adaptation.

**Fig 8:**
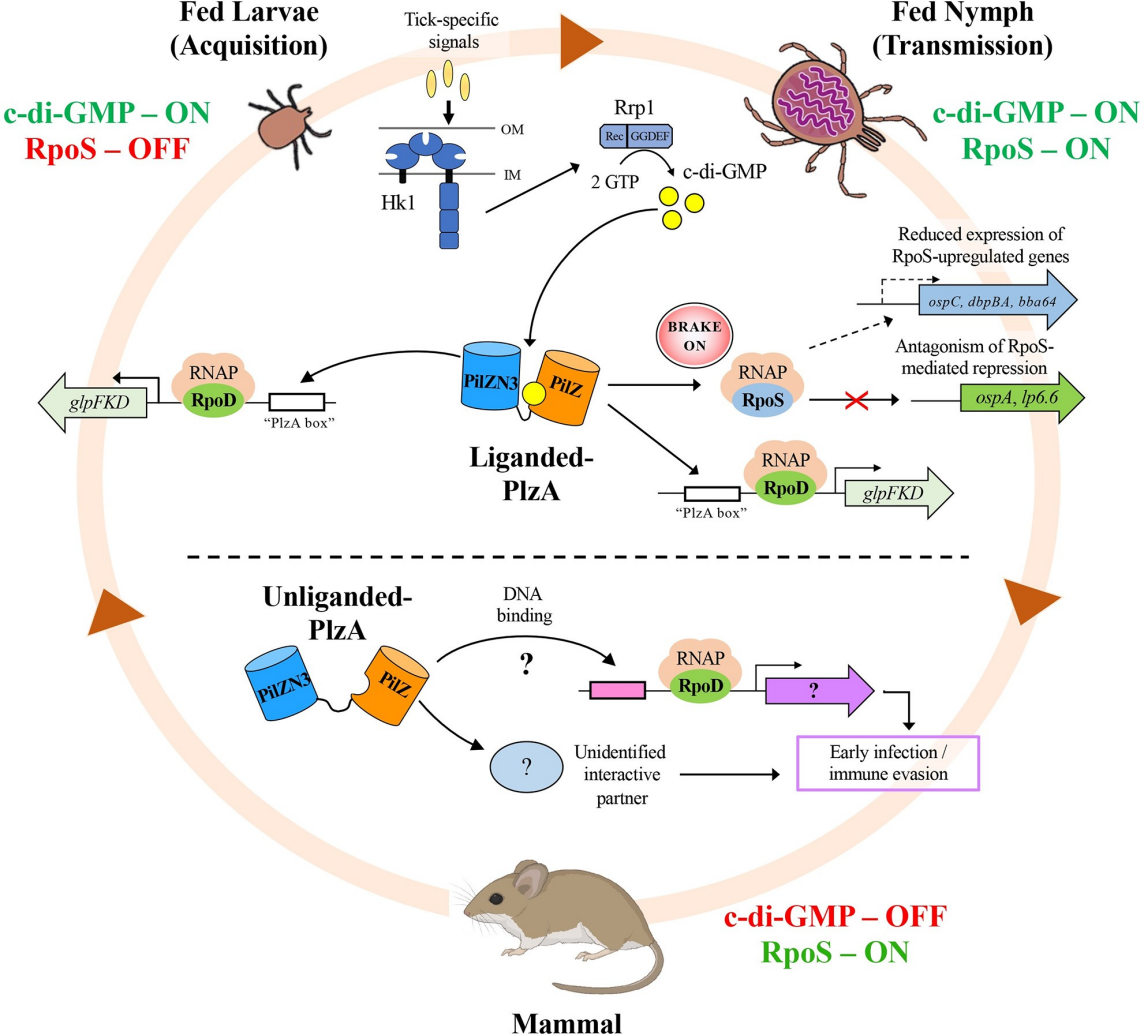
Proposed model for the function(s) of liganded- and unliganded-PlzA throughout the enzootic cycle. Global gene expression by *B*. *burgdorferi* throughout the enzootic cycle is modulated in large part by the ON and OFF states of two major regulatory networks–the Hk1/Rrp1 two component system and the RpoN/RpoS pathway [3,20,42]. During acquisition, exogenous as yet unidentified small molecules generated during tick feeding activate an Hk1-dependent signaling cascade that culminates in phosphorylation of Rrp1 and synthesis of c-di-GMP [37–39,41–46]. Binding of c-di-GMP by PlzA (liganded-PlzA) promotes transcription of the *glpKFD* operon and other tick-phase genes, presumably by binding to an upstream “PlzA box” [38,41,42,53]. At the same time, transcription of *rpoS* by the BosR/Rrp2/RpoN complex ceases and spirochetes transition from an RpoS-ON to -OFF state [3,20,42]. To expedite the switch from an RpoS-ON to -OFF state, liganded-PlzA interferes with transcription by residual RpoS allosterically by, in essence, acting as a ‘brake’ on RNAP-RpoS holoenzyme. The absence of *rpoS*/RpoS within fed larval midguts allows for unfettered expression of RpoD-dependent tick-phase genes (*i*.*e*., *glps*, *ospA*, *lp6*.*6*) that are repressed by RpoS in mammals [36,42,55,61]. During transmission, c-di-GMP signaling also is activated (*i*.*e*., ON) in response to tick-specific signals, again allowing for expression of the *glp* operon and other tick-phase genes that are upregulated by liganded-PlzA [36,38,41,42,45,53]. In contrast to acquisition, however, during transmission, BosR/Rrp2/RpoN-dependent transcription of *rpoS* is induced [3,20,42], making this the only point within the enzootic cycle when both the c-di-GMP and RpoN/RpoS pathways are ON. Based on findings presented herein, we postulate that the ‘RpoS-brake’ function of liganded-PlzA prevents RpoS-mediated repression of tick phase genes, which are required for survival within fed midguts, while at the same time allowing transcription of RpoS-upregulated genes required for transmission (*i*.*e*., *bba64*) and/or early infection (*i*.*e*., *ospC*, *dbpBA*) [36,82,95]. Once within the mammal, loss of c-di-GMP signaling (i) restores RpoS’s Gatekeeper function and (ii) enables unliganded-PlzA, working directly or indirectly *via* an unidentified interactive partner, to assume its alternative, c-di-GMP-independent virulence-related function(s).

*In vitro* and *in vivo* studies of PlzA function yield widely disparate results. *In vitro*, the consequences of PlzA deficiency are negligible (*i*.*e*., minor growth and motility defects), whereas the *in vivo* effects related to the loss of PlzA are dramatic [38,44,49,57]. As shown here and elsewhere [49,57], Δ*plzA* spirochetes are destroyed in ticks and, in mice, they exhibit marked attenuation of infectivity; importantly, neither phenotype can readily be related to the modest motility defect observed *in vitro* by others [49,57]. As noted by Novak *et al*. [44], *B*. *burgdorferi* mutants defective in motility (*e*.*g*., *cheA2* and *pdeA*) not only survive but replicate exponentially within feeding ticks [50,83]. Rather, the bulk of available evidence indicates that lysis of Δ*hk1* and Δ*rrp1* spirochetes during the blood meal is due to decreased expression of permeases for the uptake and utilization of alternative carbon sources (*e*.*g*., glycerol, N-acetylglucosamine (NAG) and chitobiose) with consequent inability to support both energy generation and cell envelope biogenesis [16,41,42,45,54]. Lysis of Δ*plzA* and *plzA-R145D* strains under these same conditions implies that these transcriptional effects of c-di-GMP are, at least in part, PlzA-dependent. Other investigators previously have raised the possibility that PlzA function in mammals might be c-di-GMP-independent [44,52,53,57]. We confirmed this by demonstrating that the virulence of the *plzA-R145D* strain is comparable to wild-type. In other bacteria, the regulatory effect of PilZ domain proteins on the flagellar motor requires c-di-GMP [84–86]; results with the *plzA-R145D* strain also argue that PlzA’s role in borrelial virulence is unrelated to motility. While non-canonical PilZ domain proteins (*e*.*g*., *Vibrio cholerae* PlzB and *Xanthamonas campestris* Xcc6021) that are unable to bind c-di-GMP have been linked to virulence [64,68,87], to our knowledge, PlzA is the first example of a c-di-GMP biosensor with dual functionality. Because the effector functions of c-di-GMP-liganded and -unliganded PlzA, respectively, are strictly segregated to the tick and mammalian stages of the enzootic cycle (Fig 8), it seems almost certain that they reflect discrete interaction partners and downstream effector mechanisms [49,53,57].

In independent studies, Sze *et al*. [45] and He *et al*. [52] reported that inactivation of *rrp1* and *plzA*, respectively, resulted in reduced levels of BosR *in vitro*, leading to the conclusion that reduced transcription of *rpoS* is responsible for the virulence defect of the Δ*plzA* mutant. Our data are not in accord with this reasoning, as we observed no effect on *rpoS*/RpoS levels either *in vitro* or in DMC-cultivated Δ*plzA* spirochetes. Moreover, we detected comparable levels of *rpoS/*RpoS in *wt*, Δ*rrp1* and *cDGC* strains in DMCs ([42] and herein). Collectively, these results indirectly confirm that the levels of RpoS are not adversely affected by loss of either liganded- or unliganded-PlzA and that, consequently, one must look elsewhere to understand how PlzA interfaces with RpoS. They also strongly infer that the early infection defect of Δ*plzA* spirochetes is both RpoS- and c-di-GMP-independent and, by extrapolation, that PlzA promotes mammalian host-adaptation *via* a mechanism(s) that is extrinsic to the RpoN/RpoS pathway. Although this RpoS-/c-di-GMP-independent function of PlzA is cryptic at present, one can glean insights into its general features from three facets of the Δ*plzA* murine phenotype uncovered herein: (**i**) The Δ*plzA* defect is overcome by a small subpopulation of organisms; in 2 of 10 C3H/HeJ mice inoculated with the Δ*plzA* strain cultivated *in vitro*, the mutant spirochetes survived the inoculation, disseminated and persisted in metastatic sites. Consistent with these findings, Pitzer *et al*. [49] found that they could increase the proportion of mice with disseminated infection by increasing the Δ*plzA* inoculum. (**ii**) The Δ*plzA* defect can be bypassed to a substantial degree if the mutant is host-adapted in DMCs prior to inoculation. (iii) The Δ*plzA* defect involves, at least in part, evasion of adaptive immunity since Δ*plzA* infectivity is substantially greater in *scid* mice. A body of evidence indicates that, once inoculated, spirochetes must overcome stochastic bottlenecks created by host barriers to cause systemic infection [88,89]. Unliganded-PlzA appears to participate in one or more regulatory pathways that increase the probability that an infecting population will contain a sufficient number of organisms with the ‘appropriate’ transcriptional profile to surmount these bottlenecks.

Fig 8 presents our working model for how gene regulation by PlzA, c-di-GMP and RpoS interdigitates to sustain *B*. *burgdorferi* within its dual-host lifecycle. In mammals, when c-di-GMP signaling is normally OFF, one sees RpoS-mediated repression of tick-phase genes [36,55,56,62,90]. When this ‘gatekeeper’ function of RpoS was first noted [55,61], predating the discovery of c-di-GMP signaling in *B*. *burgdorferi* [37–39,41,43,46], we postulated that RpoS-mediated repression was induced by mammalian host-specific signals [55,61]. However, two subsequent, closely related lines of evidence recently led us to propose an alternative hypothesis, namely, that antagonism of RpoS within feeding ticks is alleviated in the mammal by the cessation of c-di-GMP synthesis [36,42,82]: (**i**) RpoS-mediated repression of tick-phase genes does not occur concurrently with the RpoS-ON state in feeding nymphs and (**ii**) in engorged nymphs, Δ*rpoS* organisms express significantly higher levels of RpoS-repressed genes than *wt* [42,82]. Herein, we assessed this counterintuitive idea by engineering a *B*. *burgdorferi* strain (*cDGC*) that constitutively synthesizes c-di-GMP at levels comparable to those produced by *wt* strains. The results were striking and, once again, underscore the dichotomy between *in vitro* and *in vivo* gene expression by *B*. *burgdorferi*. *In vitro*, ‘ectopic’ expression of c-di-GMP had no discernible effect on the RpoN/RpoS pathway, while *in vivo* we saw a dramatic PlzA-dependent dampening of RpoS activity that affected both RpoS-upregulated and RpoS-repressed gene products. Our finding that this antagonism occurred without diminution of either *rpoS* transcript or protein, suggesting that liganded-PlzA directly or indirectly interacts with RNA polymerase-RpoS holoenzyme (RNAP-RpoS). In conjunction with these new insights, one can envision how liganded-PlzA could act as a ‘brake’ for RpoS-dependent gene regulation in ticks. During acquisition, transcription of *rpoS* by the Rrp2/BosR/RpoN complex is quickly shut OFF in the midgut in response to unknown signals [16,20]. Conceivably, to expedite the switch from an RpoS-ON to -OFF, liganded-PlzA also could interfere with transcription by residual RNAP-RpoS holoenzyme allosterically, while BBD18, which is RpoS-repressed and c-di-GMP-induced [36,42], would target free RpoS for proteolytic degradation [91–94]. During transmission, partial restriction of RNAP-RpoS holoenzyme by liganded-PlzA could enable spirochetes to sustain expression of RpoS-repressed tick phase genes (*i*.*e*., *ospA*, *glp*s) while at the same time allowing reduced expression of RpoS-upregulated genes required for transmission (*i*.*e*., *bba64*) or early infection (*i*.*e*., *ospC*, *dbpBA*) [36,82,95]. Once within the mammal, loss of c-di-GMP signaling, presumably hastened by the spirochete’s two phosphodiesterases, PdeA and PdeB [44,48,50], removes impedance of the RpoS gatekeeper and enables unliganded-PlzA to assume its alternative, c-di-GMP-independent function(s) either directly or indirectly *via* an as yet unidentified interactive partner.

The crystal structures of MrkH [66], FlgZ [67], and PlzD [96] in their liganded and unliganded states show that c-di-GMP binding causes large spatial rotations in the N- and C-terminal domains and reorganization with their inter-domain linkers, with minimal structural changes in the individual domains. c-di-GMP-induced conformational changes in PlzA [51,57], confirmed herein by SAXS, also likely underlie this novel effector protein’s dual functionality in ticks and mice. In its liganded state, PlzA forms a stable, compact protein, while the apo-protein is partially unfolded and/or significantly flexible; our analysis further suggests that apo-PlzA requires an interaction partner to promote structural stabilization and functionality. The extended length of the PlzA linker region likely contributes to even greater flexibility between its PilZN3 and PilZ domains [51]. Reorientation of the three α-helices of the PilZN3 domain in liganded PlzA would create a new interface for interactions distinct from those of the unliganded protein. Expression data for the *glp* operon indicate that liganded-PlzA directly or indirectly promotes expression of this RpoS-repressed locus by RNAP-RpoD (σ^70^) [42,53]; this could occur by two possible mechanisms (Fig 8). One, analogous to MrkH, is by DNA binding as a transcription factor [66,97]. As with MrkH [66,97], the surface electrostatics of our PlzA model indicate large positively charged regions, including major grooves in N- and C-terminal β-barrels. The other, as proposed by Zhang *et al*. [53], is by allosteric interaction with RNAP-RpoD. The possibility that PlzA can act as a positive or negative regulator for RNAP-RpoD and RNAP-RpoS holoenzymes, respectively, presents a unifying and, therefore, appealing mechanism for PlzA’s transcriptional effects.

Efficient migration between the vector and reservoir hosts is essential for perpetuation of *B*. *burgdorferi* in nature [3,7–9]; once within a reservoir or incidental human host, motility becomes critical for dissemination and tissue invasion [98–100]. In other bacteria, there is an inverse relationship between intracellular c-di-GMP concentrations and motility, with low levels promoting motility and high levels stimulating adherence and biofilm formation [76,101]. In Gram-negatives, YcgR orthologs acts as a ‘clutch’ to slow flagellar rotation in response to c-di-GMP by directly interacting with the flagellar motor at the rotor-stator interface [76,86,102]. Results presented herein show that *B*. *burgdorferi* deviates from this motility control paradigm. Comparison of the swimming behaviors of *wt*, Δ*rrp1* and *cDGC* strains *in vitro* revealed that elevated c-di-GMP exerts a potent inhibitory effect on motility; however, as shown here and elsewhere [49,57], inhibition by c-di-GMP appears to be largely independent of PlzA given that neither Δ*plzA* nor *plzA-R145D* exhibited discernably aberrant motility in BSK-II. Along similar lines, using a Δ*pdeA*Δ*plzA* double mutant, Pitzer *et al*. [49] demonstrated that elevated c-di-GMP regulates motility in *B*. *burgdorferi* by a PlzA-independent mechanism. Thus, *B*. *burgdorferi* appears to contain an as yet unidentified c-di-GMP-responsive regulator of motility that does not contain a recognizable PilZ domain. The broader implication is that the spirochete has, at least in part, separated c-di-GMP control of motility from PlzA-mediated gene expression which is needed for survival during the acquisition and transmission blood meals. Previously, we reported that spirochete transmission during the nymphal blood meal is biphasic, occurring initially *via* a non-motile, replicative process termed ‘adherence-mediated migration’ followed by an invasive, motile phase [13]. In support of this theory, we demonstrated that the contents of engorged midguts inhibit *B*. *burgdorferi* motility, now attributable to luminal tick factor(s) that stimulates synthesis of c-di-GMP via activation of the Hk1 sensor. Presumably, in the small number of spirochetes that reach the midgut basement membrane, these stimulatory cues diminish, allowing c-di-GMP levels to drop low enough to restore motility. Spirochetes lacking RpoS survive within the nymphal midgut, indicating indirectly that Hk1/Rrp1 pathway is active, but remain confined within the luminal space [82]. Thus, the ON/OFF state of the RpoN/RpoS pathway likely determines whether spirochetes colonize or penetrate the midgut epithelium in concert with precise, spatiotemporal regulation of c-di-GMP production [42,82].

## Methods

### Ethics statement

All experiments involving animals conducted at UConn Health were performed in accordance with The Guide for the Care and Use of Laboratory Animals (8th Edition) [103] using protocols reviewed and approved by the UConn Health Institutional Animal Care and Use Committee [Animal Welfare Assurance (AWA) number A347-01].

### Bacterial strains and culture conditions

S1 Table contains detailed descriptions of strains and plasmids used in these studies. *Escherichia coli* Top10 (Life Technologies, Grand Island, NY) or Stellar (TaKaRa, Mountain View, CA) strains were maintained in Lysogeny broth (LB) or LB agar supplemented with the appropriate antibiotics (ampicillin, 100 μg/ml; spectinomycin, 100 μg/ml; kanamycin, 100 μg/ml; and/or gentamycin, 5 μg/ml). *B*. *burgdorferi* strains were maintained in Barbour-Stoenner-Kelly (BSK)-II medium [104] supplemented with 6% rabbit serum (Pel-Freeze Biologicals, Rogers, AR); when appropriate, antibiotics were added (kanamycin, 400 μg/ml; streptomycin, 100 μg/ml; gentamycin, 50 μg/ml). Plasmid content of *B*. *burgdorferi* strains was monitored as previously described [105,106]. Temperature-shifts were performed as previously described [107]. For growth curves, *B*. *burgdorferi* cultures were inoculated in quadruplicate at a starting density of 1 × 10^3^ spirochetes/ml in BSK-II containing appropriate antibiotics and grown at 37°C for up to 10 days. Mammalian host-adapted spirochetes were generated by cultivation in rat peritoneal dialysis membrane chambers (DMCs) as previously described [108,109]. Spirochetes were enumerated daily by darkfield microscopy using a Petroff-Hausser counting chamber (Hausser Scientific Co., Horsham, PA). Spirochete motility was assessed by darkfield microscopy on an Olympus BX41 epifluorescence microscope using a ×100 UPlanFI objective (total magnification ×1000). Images were acquired for 10 sec per field using a QImaging Retiga R6 CCD camera (Teledyne Photometrics, Tucson, AZ).

### Routine DNA manipulation and cloning

Oligonucleotide primers used these studies are described in S3 Table. Plasmids were purified from *E*. *coli* using QIAprep spin and midi kits (Qiagen, Valencia, CA) or NucleoBond PC2000 (TaKaRa, Mountain View, CA). Bacterial genomic DNA was extracted using the Gentra Puregene Yeast/Bacteria kit (Qiagen). Except where noted, routine cloning was performed using the In-Fusion HD Cloning Plus kit (Takara Bio USA Inc., Mountain View, CA). Routine and high-fidelity PCR amplifications were performed using RedTaq (Denville Scientific, Metuchen, NJ, United States) and CloneAmp HiFi (Takara Bio USA Inc., Mountain View, CA), respectively. DNA sequencing was performed by Genewiz, Inc. (Cambridge, MA) and analyzed using MacVector v17.0.1 (MacVector, Inc., Cary, NC, United States). Oligonucleotide primers were purchased from Sigma-Aldrich (St. Louis, MO); S4 Table provides primer sequences.

### SDS-PAGE and immunoblot analyses

Whole-cell lysates were prepared from spirochetes (~2 × 10^7^ cells/lane) cultivated to late logarithmic phase at 37°C following temperature-shift were separated on 12.5% SDS-PAGE mini-gels and stained with silver as previously described [55]. For immunoblotting, whole cell lysates and/or recombinant proteins were transferred to nitrocellulose and incubated overnight with antiserum against RpoS/BB0771 [110], FlaB/BB0147 [61], GlpD/BB0243 [56], Lp6.6/BBA62 [63], OspC/BBB19 [36], OspA/BBA15 [36], or PlzA/BB0733, each diluted 1:1000–1:6000, followed by horseradish peroxidase (HRP)-conjugated secondary antibody (Southern Biotechnology Associates, Birmingham, AL) diluted 1:20,000. Anti-HA antibody (Sigma-Aldrich, St. Louis, MO) was used to track expression of Slr1143-HA. Immunoblots were developed using the SuperSignal West Pico chemiluminescence substrate (Pierce, Rockford, IL).

### Construction of *B*. *burgdorferi plzA* site-directed mutant

To distinguish between the c-di-GMP-dependent and -independent effector functions for PlzA, an arginine to aspartic acid point mutation was introduced at residue 145 (R145D) by site-directed mutagenesis [51]. Briefly, an *Age*I site was introduced into the p*bb0733*-Easy suicide vector [49] in the *plzA-bb0734* intergenic region using the QuikChange II site-directed mutagenesis kit (Agilent Technologies Inc., Santa Clara, CA) and primers BB0733AgeImut-F and BB0733AgeImut-R. A P_*flgB*_-GentR cassette [111] was inserted into the *Age*I site of p*bb0733*-Easy in the same orientation as *plzA*. A single point mutation (R145D) was introduced in the *plzA* coding region using primers BB0733-R145D-F and BB0733-R145D-R. The resulting suicide vector (p*bb0733*R145DGenta-Easy) was linearized by digestion with *Not*I, electroporated into *B*. *burgdorferi* strain B31 A3-68 Δ*bbe02* as previously described [112]. Transformants were screened for the gentamycin-resistance cassette by PCR using PlessGent-F and PlessGent-R. The R145D point mutation was confirmed by sequencing. A single transformant containing the same plasmid profile as the parent was selected for further analysis and designated *plzA-R145D*.

### Generation of *B*. *burgdorferi* strain expressing a constitutively active diguanylate cyclase

A Δ*rrp1* mutant was generated by transforming B31 5A18 NP1 [113] with pΔBB0419 [38]. A single streptomycin-resistant clone containing the same plasmid profile as the parent was selected for further analysis. A codon-optimized version of *slr1143*, encoding a constitutively active diguanylate cyclase from *Synechocystis* sp. [37] and C-terminal hemagglutinin (HA) tag, was synthesized by Genewiz (South Plainfield, NJ). *slr1143opt-HA* was fused to the *B*. *burgdorferi flaB* promoter *via* overlapping PCR using the primers listed in S4 Table. The P*flaB*-*slr1143opt-HA* cassette was cloned into the *Aat*II site of EcAG265 [106], a modified version of the cp26-based *E*. *coli*-*B*. *burgdorferi* shuttle vector pMC2498 [60] in which the promoterless *gfp* cassette has been replaced with an *Aat*II site. The resulting plasmid (EcAG284) was confirmed by sequencing and then electroporated into Δ*rrp1* as previously described [112]. Transformants were screened for *slr1143opt-HA* by PCR using primers 5’ bb0733 ORF and 3’ bb0733 ORF. A single gentamycin-resistant clone containing the same plasmid profile as the parent was selected and designated *cDGC*. To generate a Δ*plzA* strain that constitutively synthesizes c-di-GMP, the cassette encoding P*flaB*-*slr1143opt-HA* followed by GentR was sub-cloned into pUC19 containing ~1200 bp framing the *rrp1* coding region, so that P*flaB*-*slr1143opt-HA* + GentR replaced *rrp1* (pEcAG391). This plasmid was transformed into Δ*plzA* and a single gentamycin-resistant clone containing the same plasmid profile as the parent and designated Δ*plzA*+*cDGC*.

### Recombinant protein expression and purification

His-tagged PlzA-R145D was generated by site-directed mutagenesis as described above using *plzA*/pTrc-His [49] as a template. His-tagged PlzA and PlzA-R145D were overexpressed in *E*. *coli* Rosetta 2 (DE3) pRare (MilliporeSigma, Burlington, MA) and purified by nickel affinity chromatography using HisTrap Column (GE Healthcare Life Sciences Pittsburgh, PA) followed by size-exclusion chromatography using Superdex 200 10/300 GL column (GE Healthcare Life Sciences Pittsburgh, PA). Recombinant proteins were assessed for purity by SDS-PAGE followed by staining with GelCode Blue (Thermo Fisher Scientific, Waltham, MA) and/or immunoblot using HRP-conjugated anti-His antibody (ThermoFisher). Purified recombinant PlzA-His protein (40–60 μg) was used to generate polyclonal antisera in female Sprague-Dawley rats (Envigo, South Easton, MA) as previously described [108].

### Murine infection studies

Five to eight-week-old female C3H/HeJ or NOD.Cg-*Prkdc*^*scid*^/J (*scid*; Jackson Laboratories, Bar Harbor, ME) mice were needle-inoculated with 1 × 10^4^ organisms cultivated *in vitro* or from freshly harvested DMCs. At designated time points, animals were sacrificed, and blood and tissues collected for serology and culturing in BSK-II containing *Borrelia* antibiotic cocktail (0.05 mg/ml sulfamethoxazole, 0.02 mg/ml phosphomycin, 0.05 mg/ml rifampicin, 0.01 mg/ml trimethoprim and 2.5 μg/ml amphotericin B). Cultures were examined weekly by dark-field microscopy for up to 4 weeks. Seroconversion was determined by immunoblotting *B*. *burgdorferi* whole cell lysates with infected mouse serum, diluted 1:1000, followed by incubation with HRP-conjugated secondary antibody (Southern Biotechnology Associates, Birmingham, AL) diluted 1:20,000 and detection using SuperSignal West Pico chemiluminescence substrate (Pierce, Rockford, IL).

### Tick experiments

Pathogen-free *Ixodes scapularis* larvae were purchased from Oklahoma State University Tick Rearing Facility (Stillwater, OK). Immersion feeding of naïve larvae was performed as previously described [90] using the method established by Policastro *et al*. [114]. Pools of 10 replete larvae per strain were processed for DNA extraction and semi-solid phase plating. A separate pool of 10 larvae was processed for immunofluorescence using FITC-conjugated anti-*Borrelia* antibody (Kirkegaard and Perry Laboratories, Gaithersburg, MD) as previously described [90]. Replete larvae used to assess borrelial gene expression were infected by whole body infestation of needle-inoculate mice as previously described [90]. To assess gene expression during transmission, infected *I*. *scapularis* nymphs were fed for at least 72 h on naïve C3H/HeJ mice as previously described [90]. Fed larvae (~100–150 per pool) and nymphs (~20–25 per pool) were crushed into TRIzol reagent (Invitrogen) and stored at -80°C until RNA was extracted.

### qRT-PCR

Total RNA was isolated from triplicate pools of replete larvae, unfed and fed nymphs, and DMC-cultivated organisms) as previously described [90]. Following DNase treatment, RNA was converted to cDNA using SuperScript III (Life Technologies), including a negative control with no reverse transcriptase. cDNAs were assayed in quadruplicate using SsoAdvanced Universal SYBR (*rpoS*) or Probe (*flaB*) Mix (Bio-Rad) using the primers described in S3 Table. Transcript copy numbers were calculated using the iCycler post-run analysis software based on internal standard curves and then normalized against copies of *flaB* as previously described [90].

### Measurement of c-di-GMP levels

Cultures (50 mls) of *wt* B31 5A18 NP1, Δ*rrp1*, and *cDGC* were grown to late-logarithmic phase and cells were harvested for ethanol extraction as previously described [42]. Extracted supernatants were filtered through a 0.22 μm syringe-filter, concentrated via SpeedVac, and resuspended to a final volume of 0.1 ml in HPLC grade water. c-di-GMP was detected by ultraperformance liquid chromatography (UPLC) in tandem with mass spectrometry (MS) using an Acquity UPLC system coupled to an Acquity TQD mass spectrometer (Waters Corporation, Milford, MA). The separation of c-di-GMP was achieved using a High Strength Silica (HSS) reversed-phase UPLC column. Briefly, the eluent system was composed of 0.1% formic acid in water (pH 2.9) (eluent A) and 0.1% formic acid in acetonitrile (eluent B). 98% eluent A was held for 0.5 min followed by a gradient to 100% eluent B in 4 min, held for 0.5 min, then switched back to 98% eluent A at a flow rate of 0.4 ml/min. An Acquity HSS T3 column (2.1 by 100 mm; 1.8 μm particle size; Waters) was used with a sample injection volume of 10 ul. The column and autosampler were maintained at 35°C and 20°C, respectively. Detection of c-di-GMP was performed in electrospray ionization (ESI) negative-ion mode using the multiple-reaction monitoring mode. For ESI-MS/MS analysis, the following ion transition, cone voltage (CV), and collision energy (CE) were used: c-di-GMP m/z 689.1 (precursor ion) and 150.0 (product ion); CV, 66 V; and CE, 56 eV. The ESI capillary voltage was 3 kV, the source temperature was set at 150°C, and the desolvation temperature was set at 400°C. The flow rate of the desolvation gas (N2) was set at 650 liters/h. The Waters IntelliStart software was utilized for analyte signal optimization. Statistical analysis for obtaining calibration and quantification results for c-di-GMP. was performed using Waters QuanLynx, which is included in the MassLynx software v.4.2. The concentration of c-di-GMP was calculated by interpolation of the observed analyte peak area with the corresponding calibration curve. Concentrations were determined as c-di-GMP (nmol/μg) of wet cell pellet weight.

### c-di-GMP binding assay

A qualitative assessment of c-di-GMP binding by PlzA and PlzA-R145D was performed using 2’-O-(N’-Methylanthraniloyl)-c-di-GMP (MANT-c-di-GMP) (Biolog Life Science Institute GmbH & Co., Germany) [58]. Recombinant PlzA and PlzA-R145D, described above, and lysozyme (negative control) were diluted to 10 μM and incubated with 5 μM MANT-c-di-GMP in a 96 well plate, 200 μl reactions for 5 min. Binding was detected by monitoring the relative increase in fluorescence of MANT-c-di-GMP (λ_ex_ = 355 nm, λ_em_ = 448 nm) in the presence and absence of protein using a SpectraMax M2 spectrofluorometer (Molecular Devices, USA).

### Small angle X-ray scattering (SAXS) data acquisition and analysis, structural modeling, and c-di-GMP docking

Prior to SAXS, recombinant PlzA (135, 67 and 33 μM) was incubated with or without 270 μM of c-di-GMP in 500 mM NaCl and 20 mM Na_2_HPO_4_ (pH 7.4)_._ SAXS data were acquired on the Bio-SAXS beamline BL4-2 at the Stanford Synchrotron Research Laboratory using a Rayonix MX225-HE CCD detector. All scattering data (to a maximum *q* of 0.5 Å^−1^) were collected at a wavelength of 1.3 Å for ten consecutive 2-second exposures. Results from the buffer alone, with or without c-di-GMP, were subtracted from the liganded and unliganded scattering, respectively. Data were analyzed using the ATSAS package [115]. Kratky plots and radii of gyration (*R*_g_), extrapolated from the Guinier region of the Guinier plot, were computed using PRIMUS [116]. Scattering curves for liganded-PlzA were scaled and merged in *PRIMUS* primarily using the low q data for PlzA at 33 μM and the high q range data for PlzA at 67 μM. Unliganded-PlzA was analyzed using data set collected from PlzA at 67 μM only. P(r) functions were calculated using GNOM and ambiguity scores by AMBIMETER [117]. *Ab initio* shape determination was performed using DAMMIN [118] followed by DAMAVER [119]. Twenty three-dimensional models of PlzA were predicted using trRosetta [70], I-TASSER [71,72] and Swiss-Model [73] (S4 Table). Theoretical scattering curves were computed from different structural models and compared to the experimental scattering curves using FoXS [120]. The best-fitted model was refined by normal mode analysis from SAXS data using SREFLEX [74]. Coordinates for c-di-GMP were extracted from the crystal structure of *Vibrio cholerae* VCA0042/PlzD (PDB 2RDE) [96]. HADDOCK v2.2 [75] was used to dock c-di-GMP into the SREFLEX refined PlzA model. In the docking protocol, PlzA residues R145, R149, D182, A184 and G187 were designated as active residues to apply distance restraints. Superimposition of the PlzA model into the SAXS envelope structure was performed by SUPCOMB [121]. PyMOL Molecular Graphics System v2.3.2 (Schrödinger, LLC, New York, NY) was used for structure visualization, calculation of RMSD and surface electrostatics, and image rendering.

### Statistical analysis

Growth curves were compared using the CGGC permutation test [122], with 1000 permutations. All other pairwise comparisons were evaluated by unpaired Student’s *t-*test with two-tailed *p* values and a 95% confidence interval using Prism v8.4.3 (GraphPad Software, San Diego, CA).

## Supporting information

S1 Table Bacterial strains and plasmids used in these studies. (DOCX)

S2 Table Constitutive synthesis of c-di-GMP abrogates virulence of *B*. *burgdorferi* in mice. (DOCX)

S3 Table Oligonucleotide primers used in these studies. (DOCX)

S4 Table Comparison of the liganded experimental SAXS data to modeled PlzA structures. (DOCX)

S1 Fig A single point mutation within the canonical C-terminal PilZ domain abrogates binding of c-di-GMP by PlzA. A qualitative assessment of c-di-GMP binding by recombinant PlzA and PlzA-R145D His-tagged proteins was performed in triplicate using 5 μM 2’-O-(N’-Methylanthraniloyl)-c-di-GMP (MANT-c-di-GMP) [58] and 10 μM PlzA and PlzA-R145D proteins. Lysozyme (10 μM) was used as a negative control and MANT-c-di-GMP alone was used as a reference fluorescence. (**A**) Samples were excited at 355 nm and emission was measured between 400 to 500 nm at 5 nm intervals using a SpectraMax M2 spectrofluorometer (Molecular Devices, USA). Each point represents the average fluorescence at each interval following background subtraction (buffer alone). (**B**) Bars represent the changes in fluorescence (ΔF) in arbitrary units (au) for PlzA, PlzA-R145D and lysozyme compared to MANT-c-di-GMP alone at 450 nm (MANT λem_max_ = 448 nm) following background subtraction. Statistical significance was determined using unpaired Student’s *t*-test. Error bars indicate the mean ± standard error of the mean for three replicates. Asterisks (*) indicate *p* ≤ 0.05; ns, not significant.
(TIF)

S2 Fig Immunoblot analysis of sera collected from C3H/HeJ mice inoculated with *in vitro-*cultivated B31 A3-68 Δ*bbe02* (*wt*), Δ*plzA*, *plzA-R15D* and *plzA*comp strains two weeks post-infection. Sera (diluted 1:1000) were immunoblotted against *B*. *burgdorferi* strain B31 whole cell lysates. Wild-type and Δ*plzA* strains were compared to *plzA*comp (**A**) and *plzA-R145D* (**B**) strains in separate experiments (5 mice per strain, per experiment).
(TIF)

S3 Fig *B*. *burgdorferi* lacking Rrp1 or PlzA or expressing a PlzA-R154D show reduced growth *in vitro*. Growth curves of B31 A3-68 Δ*bbe02* (*wt*), Δ*plzA*, *plzA*comp, *plzA-R145D* and Δ*rrp1* strains (in quadruplicate) from a starting density of 1 × 10^3^ spirochetes/ml at 37°C. Statistical significance was determined by the CGGC permutation test [122]. Asterisks (*) indicate *p* ≤ 0.05; ns, not significant.
(TIF)

S4 Fig Host-adaptation prior to needle-inoculation restore infectivity of PlzA-deficient spirochetes in mice to near wild-type levels. **(A)** Whole cell lysates of freshly harvested DMC-cultivated B31 A3-68 Δ*bbe02* (*wt*), Δ*plzA*, *plzA-R145D* and *plzA*comp strains used to inoculate mice (Table 2) were separated by SDS-PAGE and stained with silver. Molecular weight markers (MWM) are shown on the left. (**B**) Immunoblot analysis of sera from mice needle-inoculated with DMC*-*cultivated B31 A3-68 Δ*bbe02* (*wt*), Δ*plzA*, *plzA-R145D* and *plzA*comp strains two weeks post-infection. Sera (diluted 1:1,000) were immunoblotted against *B*. *burgdorferi* whole cell lysates. Wild-type and Δ*plzA* strains were compared to *plzA*comp and *plzA-R145D* strains in separate experiments.
(TIF)

S5 Fig *cDGC* temperature-shifts normally *in vitro*. Whole-cell lysates from B31 5A18 NP1 wild-type (*wt*), Δ*rrp1* and *cDGC* strains *in vitro* following temperature-shift were separated by SDS-PAGE and immunoblotted with antisera against PlzA, RpoS, OspC and FlaB (loading control). Molecular weight markers (MWM) are shown on the left.
(TIF)

S6 Fig Comparative structural analysis of ‘xPilZ-PilZ’ proteins. (**A**) Conformational changes upon binding of c-di-GMP by *Pseudomonas putida* PP4397/FlgZ (PDBs: 2GJG-unliganded, 3KYF-liganded), *Klebsiella pneumoniae* MrkH (PDBs: 5KEC-unliganded, 5KGO-liganded), and *Vibrio cholerae* VCA0042/PlzD (PDBs: 1YLN-unliganded, 2RDE-liganded). The N- and C-terminal barrels are colored in salmon and yellow, respectively. The α-helices in the N-terminal degenerate PilZ-like domain and in the C-terminal PilZ domain are highlighted in light green and cyan, respectively. The RXXXR and (D/N)*h*SXXG c-di-GMP binding residues are represented with dark green sticks. c-di-GMP is shown in red. (**B**) Root-mean-square deviation (RMSD) values calculated from superimposition of PilZ domain from different xPilZ-PilZ proteins.
(TIF)

S7 Fig Experimental SAXS data. (**A**) Analytical SEC elution profile for PlzA in the presence (orange) or absence (blue) of c-di-GMP. The inset shows SEC calibration curve calculated by linear fit of known molecular weight values as a function of the measured partition coefficient (Kav). The black, orange, and blue circles show the partition coefficients of calibration standards, liganded, and unliganded PlzA, respectively. (**B**) Raw logarithmic intensity plots of SAXS data collected from liganded- (orange) and unliganded- (blue) PlzA. (**C**) The best fits of the PlzA model in A before (green line) and after (black line) SREFLEX optimization [74] to the experimental scattering data for liganded PlzA (orange dots). The indicated χ^2^ values were calculated by FoXS [120]. **(D**) SREFLEX refined PlzA model with a docked c-di-GMP molecule. The PilZN3 and PilZ barrels are colored in salmon and yellow, respectively. The α-helices in the PilZN3 and in PilZ domains are highlighted in light green and cyan, respectively, with the exception of the unique PilZN3 C-terminal α-helix, in purple. The RXXXR and DXSXXG c-di-GMP binding residues are represented with dark green sticks, and c-di-GMP is labeled and shown in orange. (**E**) Surface electrostatics of the refined model in **D** as calculated in PyMol. In addition to the positively charged surface of the c-di-GMP binding region, two other large positively charged region are circled by dashed lines. The ribbon structure in **D** is in the same orientation as the surface electrostatics representation in **E**.
(TIF)

S1 Movie Motility in liquid medium using darkfield microscopy of *wt* spirochetes at mid-logarithmic growth with total magnification ×1000. (MP4)

S2 Movie Motility in liquid medium using darkfield microscopy of Δ*rrp1* spirochetes at mid-logarithmic growth with total magnification ×1000. (MP4)

S3 Movie Motility in liquid medium using darkfield microscopy of Δ*plzA* spirochetes at mid-logarithmic growth with total magnification ×1000. (MP4)

S4 Movie Motility in liquid medium using darkfield microscopy of *cDGC* spirochetes at mid-logarithmic growth with total magnification ×1000. (MP4)
