# Supplementary material for: PlzA is a bifunctional c-di-GMP biosensor that promotes tick and mammalian host-adaptation of Borrelia burgdorferi
Source: PLoS Pathog. 2021 Jul 15;17(7):e1009725. doi: 10.1371/journal.ppat.1009725 (PMC8323883; doi:10.1371/journal.ppat.1009725)
Supplement: S1 Table — (DOCX) [file ppat.1009725.s001.docx]

**S1 Table. Bacterial strains and plasmids used in these studies.**

| ***Borrelia burgdorferi*** | | | | | |
| --- | --- | --- | --- | --- | --- |
| **Strain** | **Description** | **Plasmid(s) Missing^a^** | | **Antibiotic** | **Reference** |
| BbP1473  (*wt*) | *B. burgdorferi* strain B31 A3-68 bbe002::strep re-isolated from infected mouse tissue | lp5, lp56, cp9 | | Strep | [1,2] |
| BbP1474 (Δ*plzA*) | Δ*plzA* mutant in BbP1473 background. | lp5, lp56, cp9 | | Kan, Strep | [2] |
| BbP1477 (*plzA*comp) | P_plzA_*-plzA* complement in the BbP1474 background | lp5, lp56, cp9 | | Kan, Gent, Strep | [2] |
| BbP1494 (*plzA-R145D*) | *plzA* PilZ RxxD site directed mutant in BbP1473 background | lp5, lp56, cp9 | | Gent, Strep | This Study |
| BbAG103  (*wt*) | B31 5A18 NP1 *bbe02::kan* | lp28-4, lp56 | | Kan | [3] |
| BbAG551  (Δ*rrp1*) | Δ*rrp1* mutant in B31 5A18 | lp28-4, lp56 | | Kan, Strep | This Study |
| BbAG545  (*cDGC*) | cDGC into cp26 in BbAG155 background | lp28-4, lp56 | | Kan, Gent, Strep | This Study |
| BbAG557  (Δ*plzA*+*cDGC*) | *rrp1*::cDGC in BbP1474 background | lp5, lp56, cp9 | | Kan, Gent, Strep | This Study |
|  |  |  | |  |  |
| **Plasmids** | | | | | |
| **Designation** | **Description** | | **Antibiotic** | | **Reference** |
| p*bb0733-*R145D-Genta-Easy | Suicide vector used to insert *plzA*-*R145D* allele at native locus | | Amp, Kan, Gent | | This study |
| pD0419 | pCR2.1-*rrp1*::StrepR | | Amp, Kan, Strep | | [4] |
| EcAG265 | cp26 crossover vector with GentR and AatII cloning sites | | Amp, Gent | | [5] |
| EcAG284 | EcAG265 with P*flaB*-*slr1143opt-HA* | | Amp, Gent | | This study |
| EcAG391 | pUC19-*rrp1*::*cDGC+GentR* | | Amp, Gent | | This study |

a – Strains contain all endogenous B31 plasmids with the exception of those listed. All transformants retained their parental plasmid content.

**References**

1. Rego RO, Bestor A, Rosa PA. Defining the plasmid-borne restriction-modification systems of the Lyme disease spirochete *Borrelia burgdorferi*. J Bacteriol. 2011;193(5):1161-71.

2. Pitzer JE, Sultan SZ, Hayakawa Y, Hobbs G, Miller MR, Motaleb MA. Analysis of the *Borrelia burgdorferi* cyclic-di-GMP-binding protein PlzA reveals a role in motility and virulence. Infect Immun. 2011;79(5):1815-25.

3. Kawabata H, Norris SJ, Watanabe H. BBE02 disruption mutants of *Borrelia burgdorferi* B31 have a highly transformable, infectious phenotype. Infect Immun. 2004;72(12):7147-54.

4. Rogers EA, Terekhova D, Zhang HM, Hovis KM, Schwartz I, Marconi RT. Rrp1, a cyclic-di-GMP-producing response regulator, is an important regulator of *Borrelia burgdorferi* core cellular functions. Mol Microbiol. 2009;71(6):1551-73.

5. Groshong AM, McLain MA, Radolf JD. Host-specific functional compartmentalization within the oligopeptide transporter during the *Borrelia burgdorferi* enzootic cycle. PLoS Pathog. 2021;17(1):e1009180.
