# Supplementary material for: PlzA is a bifunctional c-di-GMP biosensor that promotes tick and mammalian host-adaptation of Borrelia burgdorferi
Source: PLoS Pathog. 2021 Jul 15;17(7):e1009725. doi: 10.1371/journal.ppat.1009725 (PMC8323883; doi:10.1371/journal.ppat.1009725)
Supplement: S2 Table — (DOCX) [file ppat.1009725.s002.docx]

**S2 Table. Constitutive synthesis of c-di-GMP abrogates virulence of *B. burgdorferi* in mice.**

|  | ***wt*** | **Δ*rrp1*** | ***cDGC*** |
| --- | --- | --- | --- |
| **Serology^1^** | 5/5 | 5/5 | 0/5 |
| **Ear^2^** | 5/5 | 5/5 | 0/5 |
| **Proximal skin** | 5/5 | 5/5 | 0/5 |
| **Distal skin** | 5/5 | 5/5 | 0/5 |
| **Tibiotarsal joint** | 5/5 | 5/5 | 0/5 |
| **Bladder** | 5/5 | 5/5 | 0/5 |
| **Heart** | 5/5 | 5/5 | 0/5 |
| **Total pos. sites^3^** | 30/30 | 30/30 | 0/30 |
| **Total infected^4^** | 5/5 | 5/5 | 0/5 |

^1^Serology is based on immunoreactivity of serum from individual mice against whole cell lysates of wild-type *B. burgdorferi* strain B31 cultivated at 37°C *in vitro*.

^2^Data represent culture positivity for the designated tissues collected from C3H/HeJ mice two weeks after inoculation with 1 × 10^4^ of isogenic wild-type (*wt*), Δ*rrp1* or *cDGC* strains cultivated *in vitro*.

^3^Total number of culture-positive tissues from all mice in the designated group.

^4^Total number of infected mice per group.
