# Supplementary material for: PlzA is a bifunctional c-di-GMP biosensor that promotes tick and mammalian host-adaptation of Borrelia burgdorferi
Source: PLoS Pathog. 2021 Jul 15;17(7):e1009725. doi: 10.1371/journal.ppat.1009725 (PMC8323883; doi:10.1371/journal.ppat.1009725)
Supplement: S3 Table — (DOCX) [file ppat.1009725.s003.docx]

**S3 Table. Oligonucleotide primers used in these studies.**

| Primer | Sequence (5’-3’) | Purpose | Reference |
| --- | --- | --- | --- |
| 733AgeImut-F | CGAAAATTTACCGGTAATATTT | generation of *plzA-R145D* | This study |
| 733AgeImut-R | AAATATTACCGGTAAATTTTCG | generation of *plzA- R145D* | This study |
| 733-R145D-F | GCAAAATCAG***GAT***ATTCATGAGAGG | generation of *plzA- R145D* | This study |
| 733-R145D-R | TCTCATGAAT***ATC***CTGATTTTGCCC | generation of *plzA- R145D* | This study |
| 5’PflaB_slr1143 | TCGGGTAGGATCCCGACGTCAGAAATAAATAATAATAATTATTTTTAATGCTATTGC | P*flaB*-*slr1143opt-HA cloning* | This study |
| 3’PflaB_slr1143 | TTTAAATTTTATCATGGAGGAATGATATATGGAAGCTAAATTA | P*flaB*-*slr1143opt-HA cloning* | This study |
| 5’slr1143 | ATGGAGGAATGATATATGGAAGCTAAATTACCTCAAAATGAA | P*flaB*-*slr1143opt-HA cloning* | This study |
| 3’slr1143 | TATCCTTATGATGTTCCTGATTATGCTTAAGACGTCATCTAGAAATTTTG | P*flaB*-*slr1143opt-HA cloning* | This study |
| 5’ bb0733 ORF | ATGCTTTTATCTAGAAAAATAAGAGATTATGG | Check plzA insertion | This study |
| 3’ bb0733 ORF | TTAATTGAAATAATCATGGATCAACATAG | Check plzA insertion | This study |
| 5 F1 rrp1 | CGACTCTAGAGGATCCGAGTTTAACCCAGAAATGGATTTTGAGGATTTGG | *rrp1*::*cDGC* cloning | This study |
| 3 F1 slrrrp1 | TTATTATTTATTTCTTGCTAATATCTCTTATTATTAAGCATTTCAATAATCTCTTCAAGAATCTT | *rrp1*::*cDGC* cloning | This study |
| 5 rrp1slr | TAAGAGATATTAGCAAGAAATAAATAATAATAATTATTTTTAATGCTATTGCTATTTGCGTTTCT | *rrp1*::*cDGC* cloning | This study |
| 3 gentslr | AATCTTCCTTGAAGCTTAAGCATAATCAGGAACATCATAAGGATATTCAGC | *rrp1*::*cDGC* cloning | This study |
| 5 slrgent | CCTGATTATGCTTAAGCTTCAAGGAAGATTTCCTATTAAGGTTGAACTTA | *rrp1*::*cDGC* cloning | This study |
| 5 F2gentrrp1 | AAGTACCGCCACCTAATTTATATTTAATAGACTTTAGTATTTATAAGTTATAGACATTCCAATAGAATCG | *rrp1*::*cDGC* cloning | This study |
| 3 F2 rrp1 | CGGTACCCGGGGATCCGCTCTAGACTTAACAATGTTGATGCATCTGTTAA | *rrp1*::*cDGC* cloning | This study |
| PlessGent-F | ATGTTACGCAGCAGCAACGATG | screening transformants | This study |
| PlessGent-R | TTAGGTGGCGGTACTTGGGTCGA | screening transformants | This study |
| PlessSpStr-F | ATGAGGGAAGCGGTGATCGCCGA | screening transformants | This study |
| PlessSpStr-R | TTATTTGCCGACTACCTTGGTGATCTC | screening transformants | This study |
| KanR532-F | CGGGCAATCAGGTGCGACAAT | screening transformants | This study |
| KanR1207-R | CCGTCAAGTCAGCGTAATGCTCTGCCAGT | screening transformants | This study |
| rpoS-F | CTTGCAGGACAAATACAAAGAGGC | qRT-PCR | [1] |
| rpoS-R | GCAGCTCTTATTAATCCCAAGTTGCC | qRT-PCR | [1] |
| flaB-F | CTTTTCTCTGGTGAGGGAGCTC | qRT-PCR | [2] |
| flaB-R | GCTCCTTCCTGTTGAACACCC | qRT-PCR | [2] |
| flaB-probe | [6FAM]CTTGAACCGGTGCAGCCTGAGCA[BHQ1] | qRT-PCR | [2] |

Restriction sites underlined.

Point mutation shown in bold italics.

**References**

1. Caimano MJ, Iyer R, Eggers CH, Gonzalez C, Morton EA, Gilbert MA, et al. Analysis of the RpoS regulon in *Borrelia burgdorferi* in response to mammalian host signals provides insight into RpoS function during the enzootic cycle. Mol Microbiol. 2007;65(5):1193-217.

2. Pal U, Li X, Wang T, Montgomery RR, Ramamoorthi N, Desilva AM, et al. TROSPA, an *Ixodes scapularis* receptor for *Borrelia burgdorferi*. Cell. 2004;119(4):457-68.
