# Supplementary material for: PlzA is a bifunctional c-di-GMP biosensor that promotes tick and mammalian host-adaptation of Borrelia burgdorferi
Source: PLoS Pathog. 2021 Jul 15;17(7):e1009725. doi: 10.1371/journal.ppat.1009725 (PMC8323883; doi:10.1371/journal.ppat.1009725)
Supplement: S4 Table — (DOCX) [file ppat.1009725.s004.docx]

**S4 Table.** **Comparison of the liganded experimental SAXS data to modeled PlzA structures.**

| **Model** | **Modelling method/server** | **χ^2^** |
| --- | --- | --- |
| Refined Model 1 | SREFLEX on model 1 | 6.66 |
| Model 1 | trRosetta^a^ | 7.13 |
| Model 2 | trRosetta^a^ | 7.18 |
| Model 3 | trRosetta^a^ | 7.56 |
| Model 4 | trRosetta^a^ | 7.60 |
| Model 5 | trRosetta^a^ | 8.01 |
| Model 6 | trRosetta^a^ | 8.12 |
| Model 7 | trRosetta^a^ | 8.23 |
| Model 8 | trRosetta^a^ | 8.51 |
| Model 9 | trRosetta^a^ | 8.84 |
| Model 10 | trRosetta^a^ | 9.51 |
| Model 11 | I-TASSER^b^ | 10.38 |
| Model 12^d^ | Swiss-model^c^ | 10.64 |
| Model 13 | I-TASSER^b^ | 11.75 |
| Model 14^e^ | Swiss-model^c^ | 12.43 |
| Model 15^f^ | Swiss-model^c^ | 12.91 |
| Model 16 | I-TASSER^b^ | 13.22 |
| Model 17 | I-TASSER^b^ | 13.46 |
| Model 18^g^ | Swiss-model^c^ | 13.92 |
| Model 19 | I-TASSER^b^ | 14.51 |
| Model 20^h^ | Swiss-model^c^ | 18.41 |

a – [1]

b – [2, 3]

c – [4]

d – Homology to PDB 3KYF, c-di-GMP bound PP4397/FlgZ from *P. putida.*

e – Homology to PDB 3KYG, c-di-GMP bound VCA0042/PlzD from *V. cholerae*.

f – Homology to PDB 5Y6F, c-di-GMP bound YcgR from *E. coli*.

g – Homology to PDB 2GJG, unbound PP4397/FlgZ from *P. putida*.

h – Homology to PDB 5KEC, unbound MrkH from *K. pneumoniae*.

**REFERENCES**

1. Yang J, Anishchenko I, Park H, Peng Z, Ovchinnikov S, Baker D. Improved protein structure prediction using predicted interresidue orientations. Proc Natl Acad Sci U S A. 2020;117(3):1496-503. Epub 2020/01/04. doi: 10.1073/pnas.1914677117. PubMed PMID: 31896580; PubMed Central PMCID: PMCPMC6983395.

2. Yang J, Yan R, Roy A, Xu D, Poisson J, Zhang Y. The I-TASSER Suite: protein structure and function prediction. Nat Methods. 2015;12(1):7-8. Epub 2014/12/31. doi: 10.1038/nmeth.3213. PubMed PMID: 25549265; PubMed Central PMCID: PMCPMC4428668.

3. Yang J, Zhang Y. I-TASSER server: new development for protein structure and function predictions. Nucleic Acids Res. 2015;43(W1):W174-81. Epub 2015/04/18. doi: 10.1093/nar/gkv342. PubMed PMID: 25883148; PubMed Central PMCID: PMCPMC4489253.

4. Waterhouse A, Bertoni M, Bienert S, Studer G, Tauriello G, Gumienny R, et al. SWISS-MODEL: homology modelling of protein structures and complexes. Nucleic Acids Res. 2018;46(W1):W296-W303. Epub 2018/05/23. doi: 10.1093/nar/gky427. PubMed PMID: 29788355; PubMed Central PMCID: PMCPMC6030848.
