# Supplementary figures and images for: PlzA is a bifunctional c-di-GMP biosensor that promotes tick and mammalian host-adaptation of Borrelia burgdorferi
Source: PLoS Pathog. 2021 Jul 15;17(7):e1009725. doi: 10.1371/journal.ppat.1009725 (PMC8323883; doi:10.1371/journal.ppat.1009725)

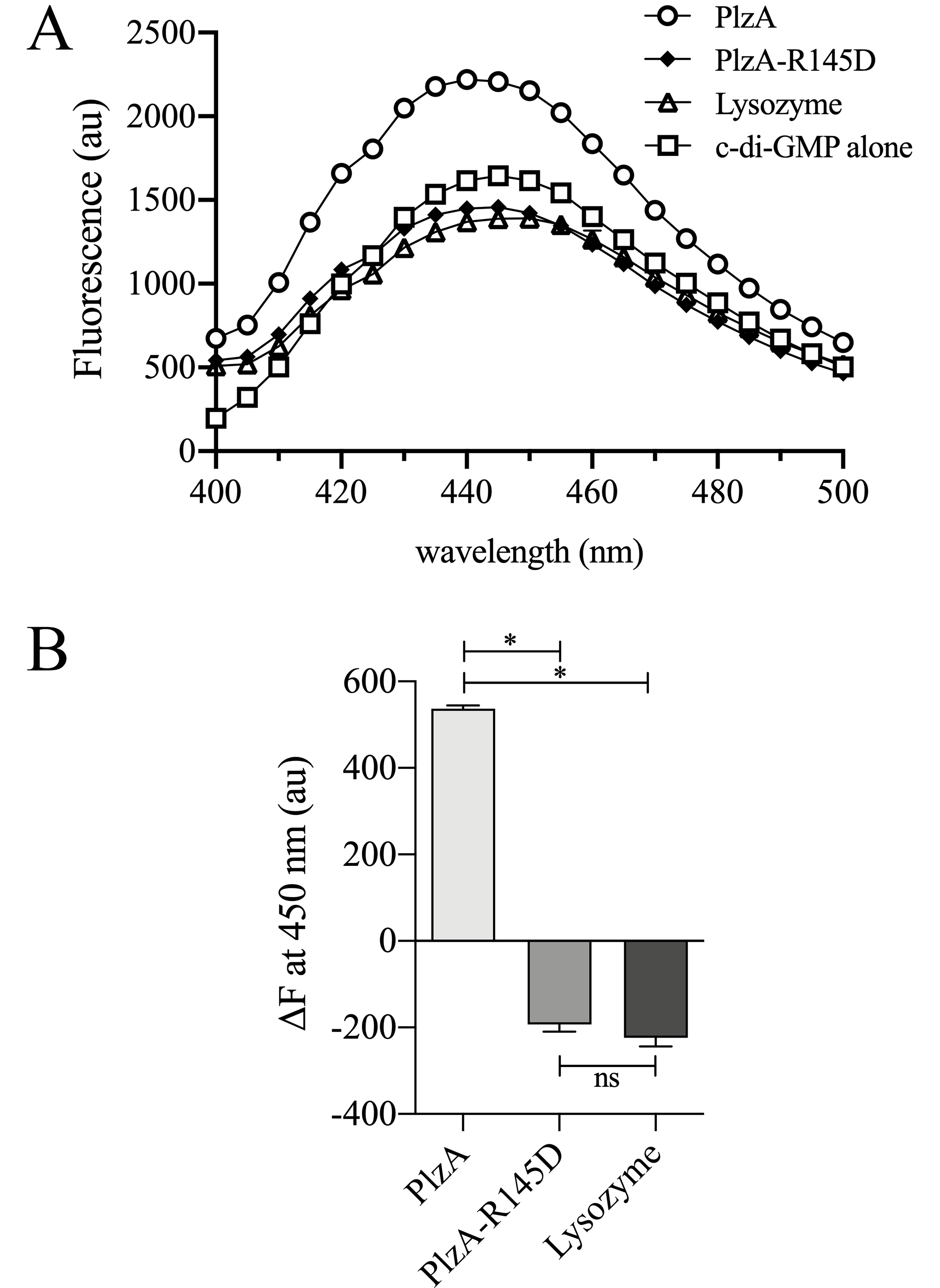

Supplement: S1 Fig — A qualitative assessment of c-di-GMP binding by recombinant PlzA and PlzA-R145D His-tagged proteins was performed in triplicate using 5 μM 2’-O-(N’-Methylanthraniloyl)-c-di-GMP (MANT-c-di-GMP) [58] and 10 μM PlzA and PlzA-R145D proteins. Lysozyme (10 μM) was used as a negative control and MANT-c-di-GMP alone was used as a reference fluorescence. (A) Samples were excited at 355 nm and emission was measured between 400 to 500 nm at 5 nm intervals using a SpectraMax M2 spectrofluorometer (Molecular Devices, USA). Each point represents the average fluorescence at each interval following background subtraction (buffer alone). (B) Bars represent the changes in fluorescence (ΔF) in arbitrary units (au) for PlzA, PlzA-R145D and lysozyme compared to MANT-c-di-GMP alone at 450 nm (MANT λemmax = 448 nm) following background subtraction. Statistical significance was determined using unpaired Student’s t-test. Error bars indicate the mean ± standard error of the mean for three replicates. Asterisks (*) indicate p ≤ 0.05; ns, not significant. (TIF) [file ppat.1009725.s005.tif]

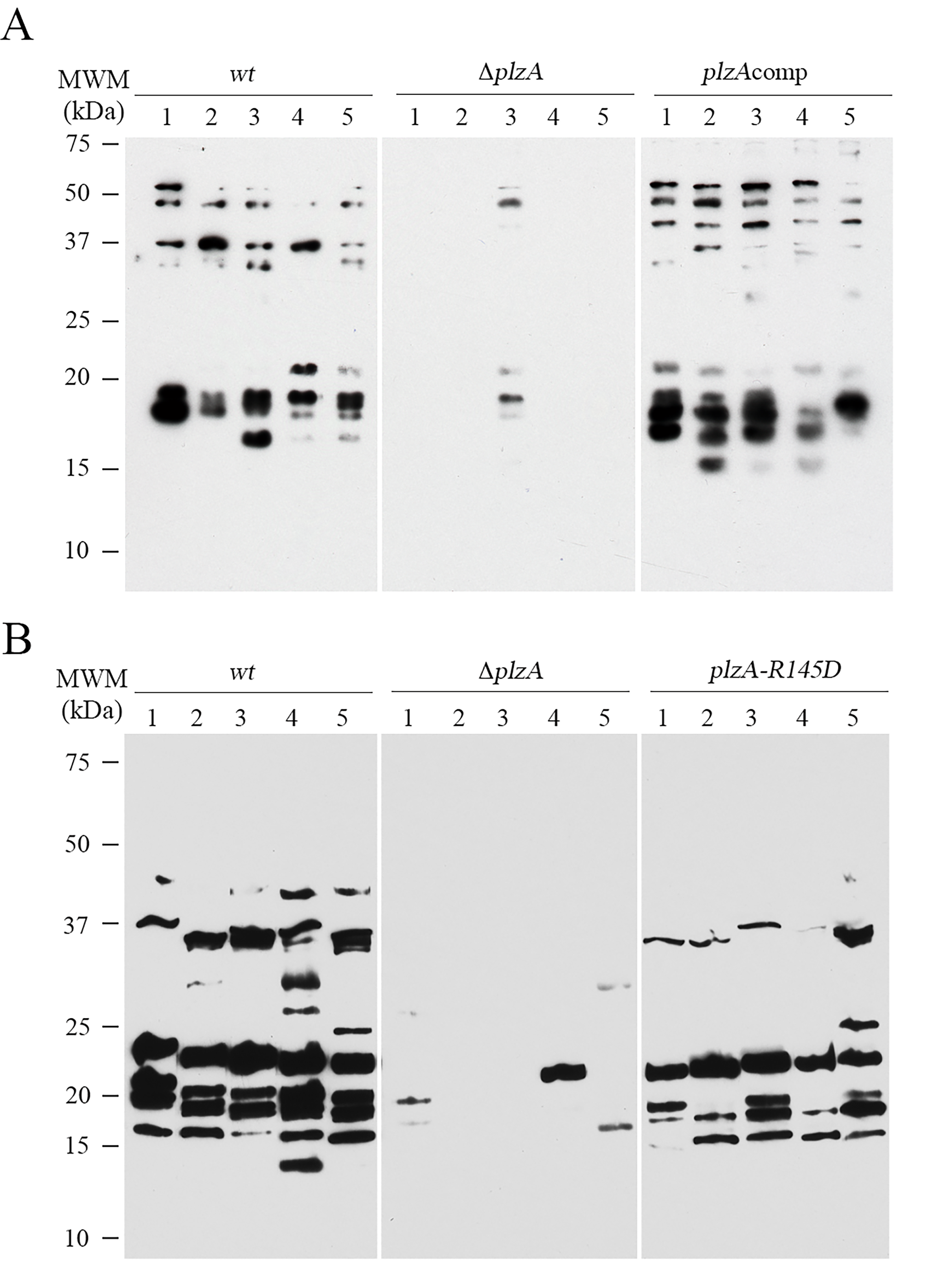

Supplement: S2 Fig — Sera (diluted 1:1000) were immunoblotted against B. burgdorferi strain B31 whole cell lysates. Wild-type and ΔplzA strains were compared to plzAcomp (A) and plzA-R145D (B) strains in separate experiments (5 mice per strain, per experiment). (TIF) [file ppat.1009725.s006.tif]

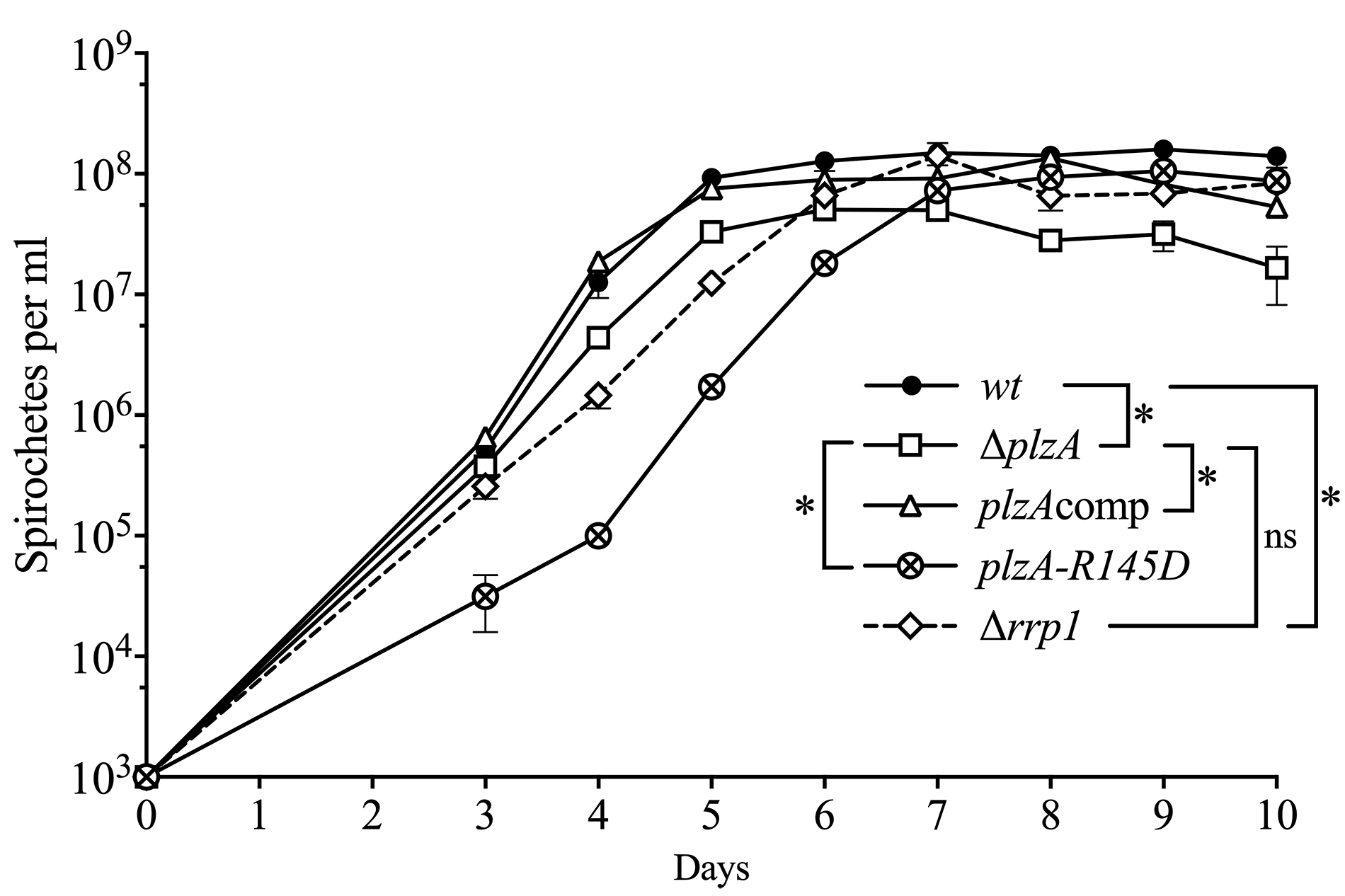

Supplement: S3 Fig — Growth curves of B31 A3-68 Δbbe02 (wt), ΔplzA, plzAcomp, plzA-R145D and Δrrp1 strains (in quadruplicate) from a starting density of 1 × 10^3^ spirochetes/ml at 37°C. Statistical significance was determined by the CGGC permutation test [122]. Asterisks (*) indicate p ≤ 0.05; ns, not significant. (TIF) [file ppat.1009725.s007.tif]

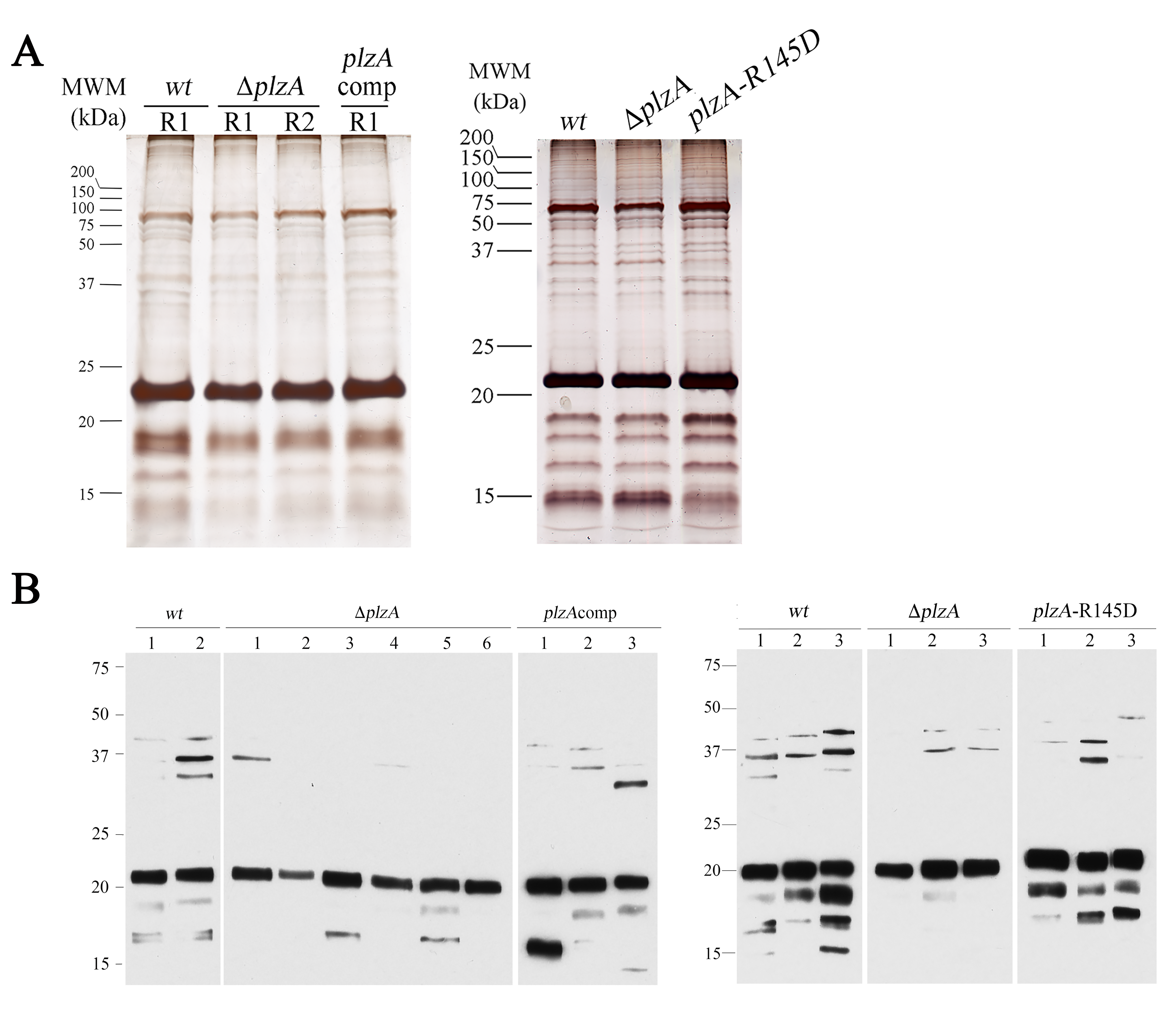

Supplement: S4 Fig — (A) Whole cell lysates of freshly harvested DMC-cultivated B31 A3-68 Δbbe02 (wt), ΔplzA, plzA-R145D and plzAcomp strains used to inoculate mice (Table 2) were separated by SDS-PAGE and stained with silver. Molecular weight markers (MWM) are shown on the left. (B) Immunoblot analysis of sera from mice needle-inoculated with DMC-cultivated B31 A3-68 Δbbe02 (wt), ΔplzA, plzA-R145D and plzAcomp strains two weeks post-infection. Sera (diluted 1:1,000) were immunoblotted against B. burgdorferi whole cell lysates. Wild-type and ΔplzA strains were compared to plzAcomp and plzA-R145D strains in separate experiments. (TIF) [file ppat.1009725.s008.tif]

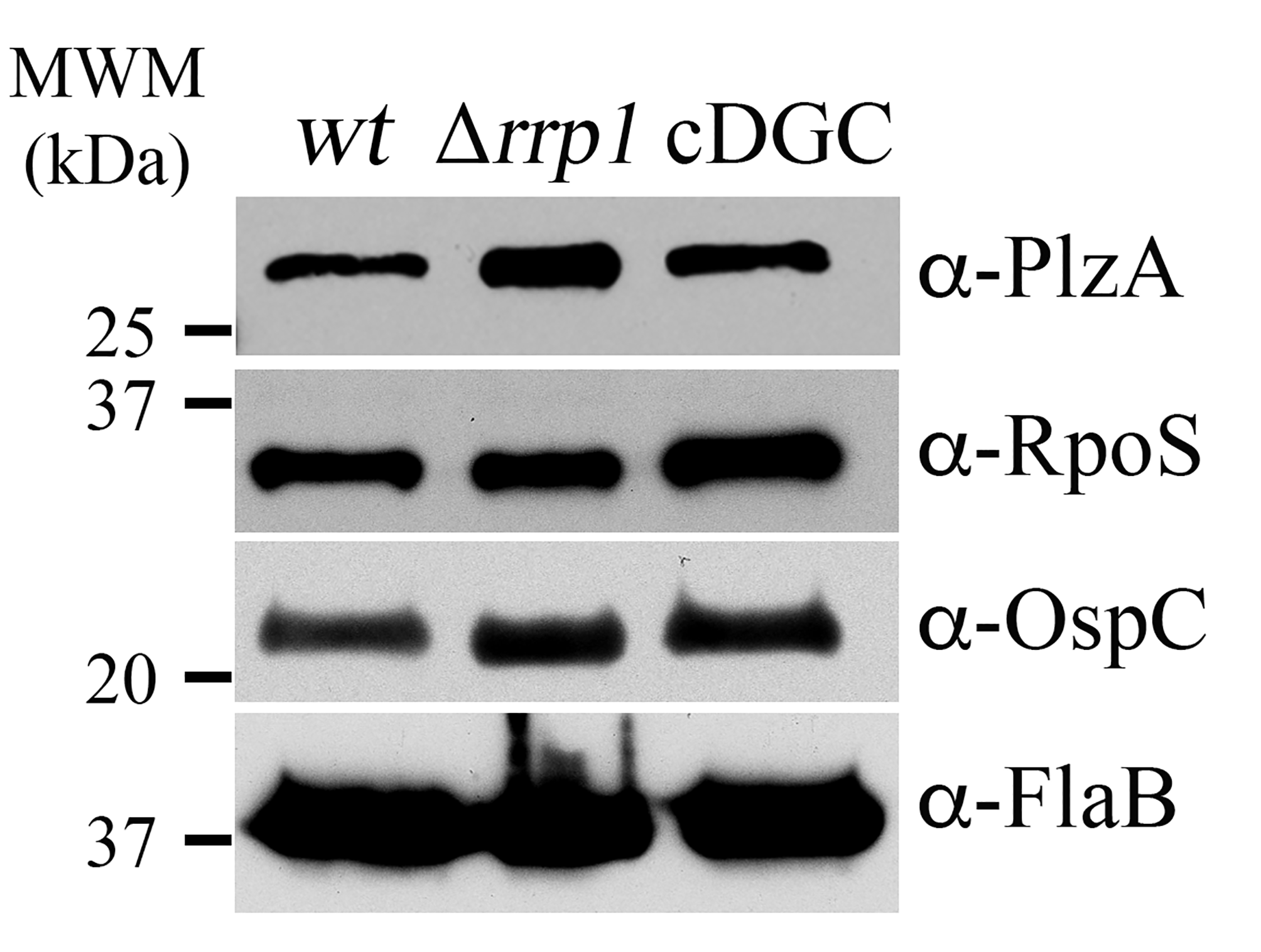

Supplement: S5 Fig — Whole-cell lysates from B31 5A18 NP1 wild-type (wt), Δrrp1 and cDGC strains in vitro following temperature-shift were separated by SDS-PAGE and immunoblotted with antisera against PlzA, RpoS, OspC and FlaB (loading control). Molecular weight markers (MWM) are shown on the left. (TIF) [file ppat.1009725.s009.tif]

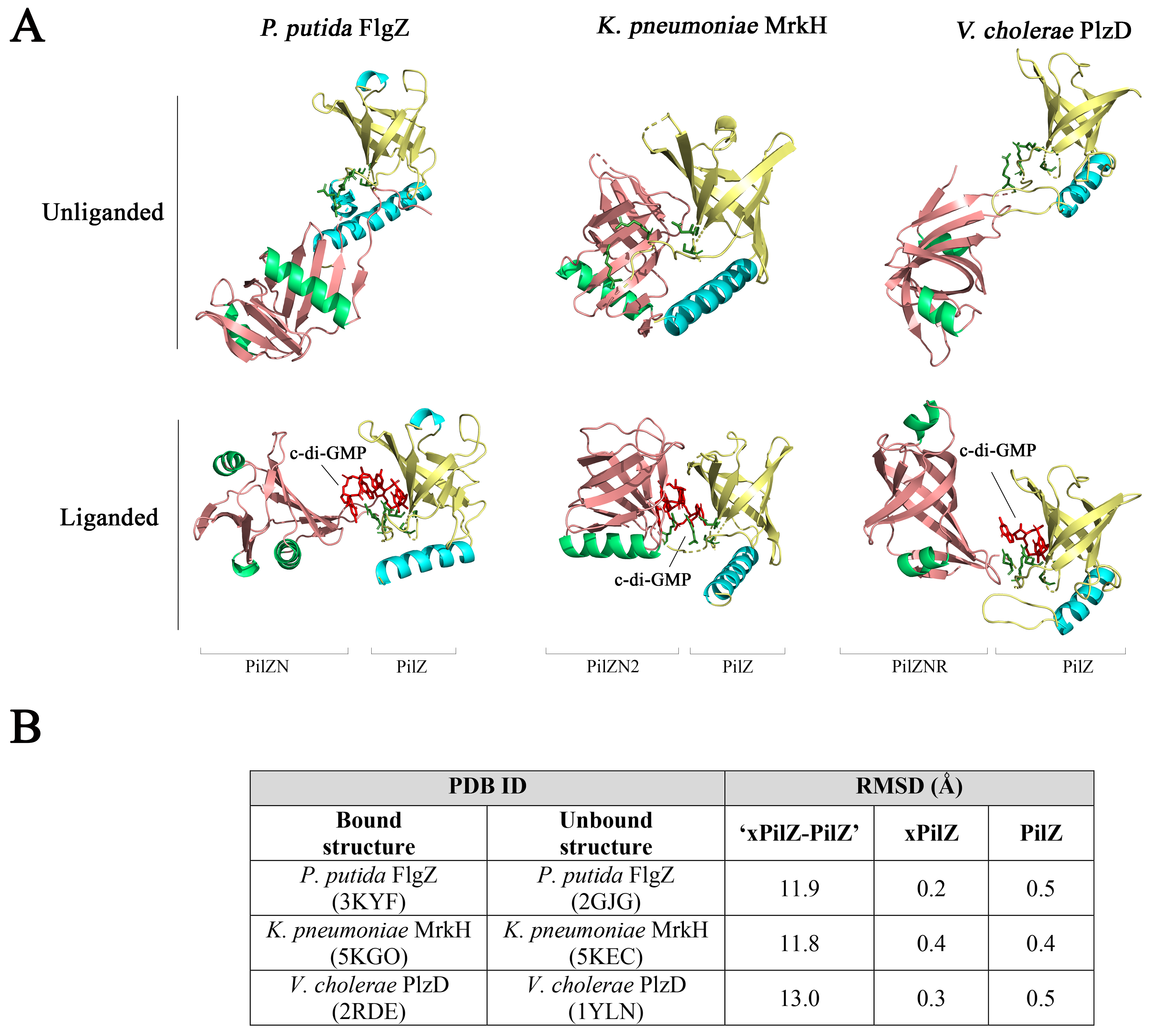

Supplement: S6 Fig — (A) Conformational changes upon binding of c-di-GMP by Pseudomonas putida PP4397/FlgZ (PDBs: 2GJG-unliganded, 3KYF-liganded), Klebsiella pneumoniae MrkH (PDBs: 5KEC-unliganded, 5KGO-liganded), and Vibrio cholerae VCA0042/PlzD (PDBs: 1YLN-unliganded, 2RDE-liganded). The N- and C-terminal barrels are colored in salmon and yellow, respectively. The α-helices in the N-terminal degenerate PilZ-like domain and in the C-terminal PilZ domain are highlighted in light green and cyan, respectively. The RXXXR and (D/N)hSXXG c-di-GMP binding residues are represented with dark green sticks. c-di-GMP is shown in red. (B) Root-mean-square deviation (RMSD) values calculated from superimposition of PilZ domain from different xPilZ-PilZ proteins. (TIF) [file ppat.1009725.s010.tif]

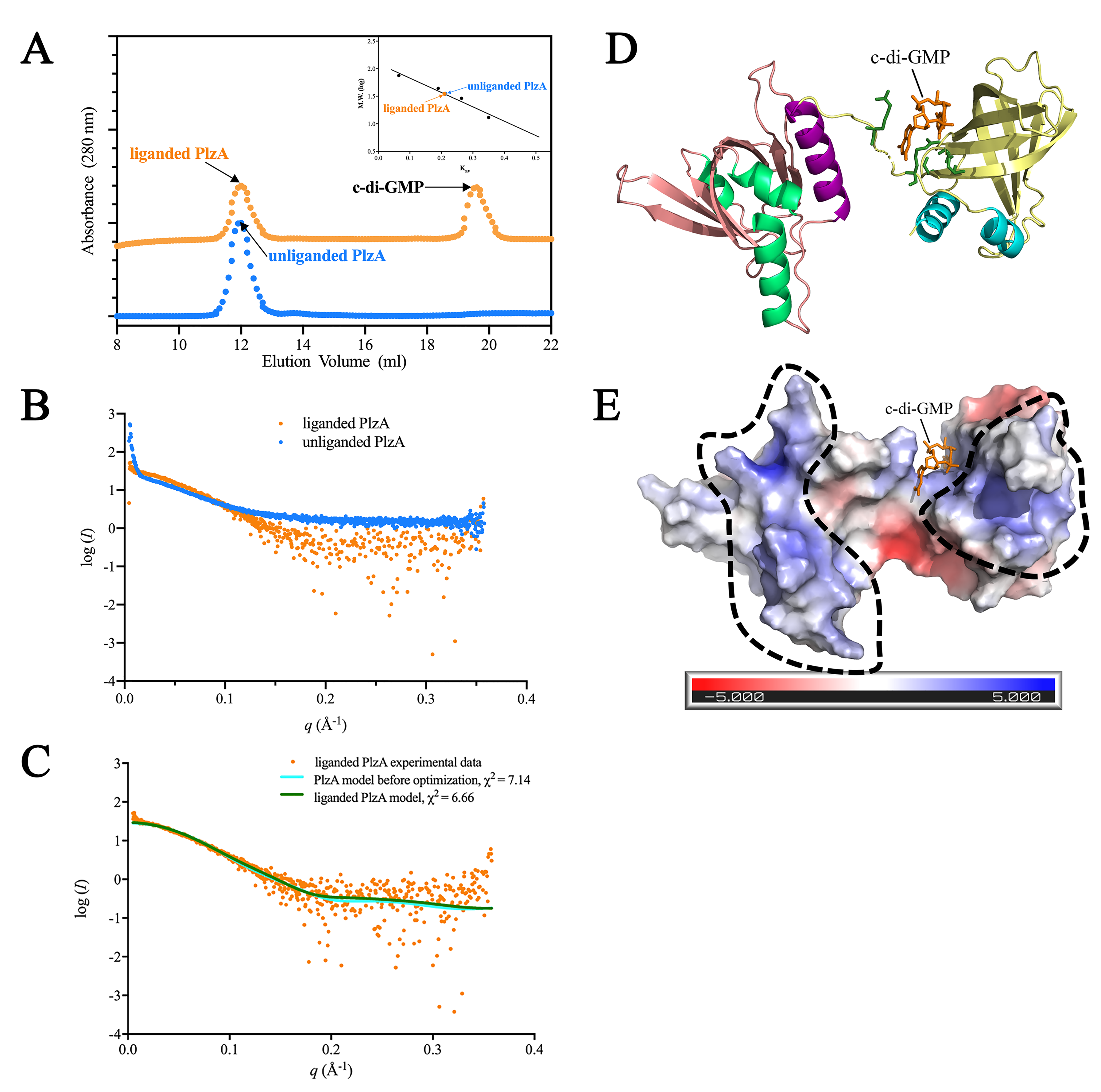

Supplement: S7 Fig — (A) Analytical SEC elution profile for PlzA in the presence (orange) or absence (blue) of c-di-GMP. The inset shows SEC calibration curve calculated by linear fit of known molecular weight values as a function of the measured partition coefficient (Kav). The black, orange, and blue circles show the partition coefficients of calibration standards, liganded, and unliganded PlzA, respectively. (B) Raw logarithmic intensity plots of SAXS data collected from liganded- (orange) and unliganded- (blue) PlzA. (C) The best fits of the PlzA model in A before (green line) and after (black line) SREFLEX optimization [74] to the experimental scattering data for liganded PlzA (orange dots). The indicated χ2 values were calculated by FoXS [120]. (D) SREFLEX refined PlzA model with a docked c-di-GMP molecule. The PilZN3 and PilZ barrels are colored in salmon and yellow, respectively. The α-helices in the PilZN3 and in PilZ domains are highlighted in light green and cyan, respectively, with the exception of the unique PilZN3 C-terminal α-helix, in purple. The RXXXR and DXSXXG c-di-GMP binding residues are represented with dark green sticks, and c-di-GMP is labeled and shown in orange. (E) Surface electrostatics of the refined model in D as calculated in PyMol. In addition to the positively charged surface of the c-di-GMP binding region, two other large positively charged region are circled by dashed lines. The ribbon structure in D is in the same orientation as the surface electrostatics representation in E. (TIF) [file ppat.1009725.s011.tif]
